# Dual effect of α-synuclein disease variants on condensate formation

**DOI:** 10.1038/s41467-026-77002-4

**Published:** 2026-09-02

**Authors:** Aswathy Chandran, Aishwarya Agarwal, Tianhao Wang, Leslie Amaral, Shiyue Chen, Susana R. Chaves, Tiago F. Outeiro, Janin Lautenschläger

**Affiliations:** 1 Cambridge Institute for Medical Research, University of Cambridge, Cambridge Biomedical Campus, The Keith Peters Building, Hills Road, Cambridge, UK; 2 University Medical Center Göttingen, Department of Experimental Neurodegeneration, Center for Biostructural Imaging of Neurodegeneration, Göttingen, Germany; 3 CBMA – Centre of Molecular and Environmental Biology, School of Sciences, University of Minho, Braga, Portugal; 4 Translational and Clinical Research Institute, Faculty of Medical Sciences, Newcastle University, Framlington Place, Newcastle Upon Tyne, UK; 5 Faculdade de Medicina e Ciências Biomédicas, Algarve Biomedical Center Research Institute (ABC-Ri), Algarve Biomedical Center (ABC), Universidade do Algarve, Faro, Portugal; 6 Department of Neuroscience, Physiology and Pharmacology, University College London, London, United Kingdom

**Keywords:** Molecular neuroscience, Parkinson's disease

## Abstract

α-synuclein is a pre-synaptic protein implicated in synucleinopathies including Parkinson’s disease, Parkinson’s disease dementia, and dementia with Lewy bodies, where it accumulates in intracellular aggregates termed Lewy bodies and Lewy neurites. Recent studies have reported that α-synuclein undergoes phase separation to form biomolecular condensates both in vitro and in mammalian cells. α-synuclein condensates are thought to contribute to disease through progressive aggregation. Here we show that specific PD-associated α-synuclein variants fail to form biomolecular condensates. We demonstrate that only two α-synuclein variants associated with disease, E46K and E83Q, enhance condensate formation. While other α-synuclein disease variants including A30G, G51D, and A53E fail to form condensates in cells. In iPSC-derived neurons, the extent of synaptic enrichment of different α-synuclein variants is linked to their propensity to undergo VAMP2-mediated phase separation. Our results emphasize that α-synuclein dysfunction may follow divergent pathways, with both increased and decreased condensate formation.

## Abstract (web summary)

This study shows that pathological α-synuclein variants have inherently different effects on cellular condensate formation. This behavior translates to differences in synaptic localization, which may have distinct pathophysiological consequences in disease.

## Introduction

Parkinson’s disease (PD) is the second most common neurodegenerative disorder, affecting millions worldwide^1,2^. It is characterized by cardinal motor symptoms of tremor, rigidity, bradykinesia, and postural instability, as well as non-motor symptoms, including autonomic dysfunction, dementia, and depression^3^. The pathophysiology of PD is multifactorial, involving genetic, environmental, and age-related factors. In PD, and other synucleinopathies like Parkinson’s disease dementia (PDD) and dementia with Lewy bodies (DLB), the protein α-synuclein (aSYN) misfolds and aggregates in neurons, forming β-sheet rich fibrillar intracellular inclusions known as Lewy bodies and Lewy neurites, hallmark features of the disease^4^. Additionally, duplication/triplication and mutations in the gene encoding aSYN (*SNCA*) cause autosomal dominant forms of early onset PD^5,6^. aSYN is a key presynaptic protein^7,8^ involved in synaptic vesicle clustering, neurotransmitter release, and maintaining synapse integrity^9^. Recent research highlights that aSYN can undergo phase separation, forming protein-enriched droplet-like structures termed biomolecular condensates^10–13^. This mechanism allows proteins to enrich in dynamic structures, which can form, but also disassemble upon physiological cues^14^. We, and others, have previously shown that aSYN phase separation is regulated by vesicle-associated membrane protein 2 (VAMP2), a SNARE complex protein responsible for synaptic vesicle fusion^12,13^. This process is modulated by the juxtamembrane domain of VAMP2 and the resulting aSYN condensates exhibit liquid-like properties^12,13^.

In this study, we explore the effect of disease-associated aSYN variants on phase separation in vitro and in different cellular environments. We find that only two aSYN variants, E46K and E83Q enhance condensate formation, while other disease variants show no or markedly reduced condensate formation in cells. We further show that in iPSC-derived neurons, aSYN variants which undergo phase separation, enrich at the synapse, however, variants which do not show phase separation, remain cytosolic. aSYN phase separation has been previously linked to subsequent aggregation and disease pathogenesis^11,15,16^, however, our findings highlight that not only increased condensate formation, but an imbalance in aSYN phase separation may contribute to its pathophysiology. This study establishes phase separation as an important molecular property of aSYN, wherein both increased or decreased levels of condensate formation can result in redistribution of aSYN at the synapse, eventually contributing to disease.

## Results

### aSYN disease variants influence condensate formation

**Fig. 1:**
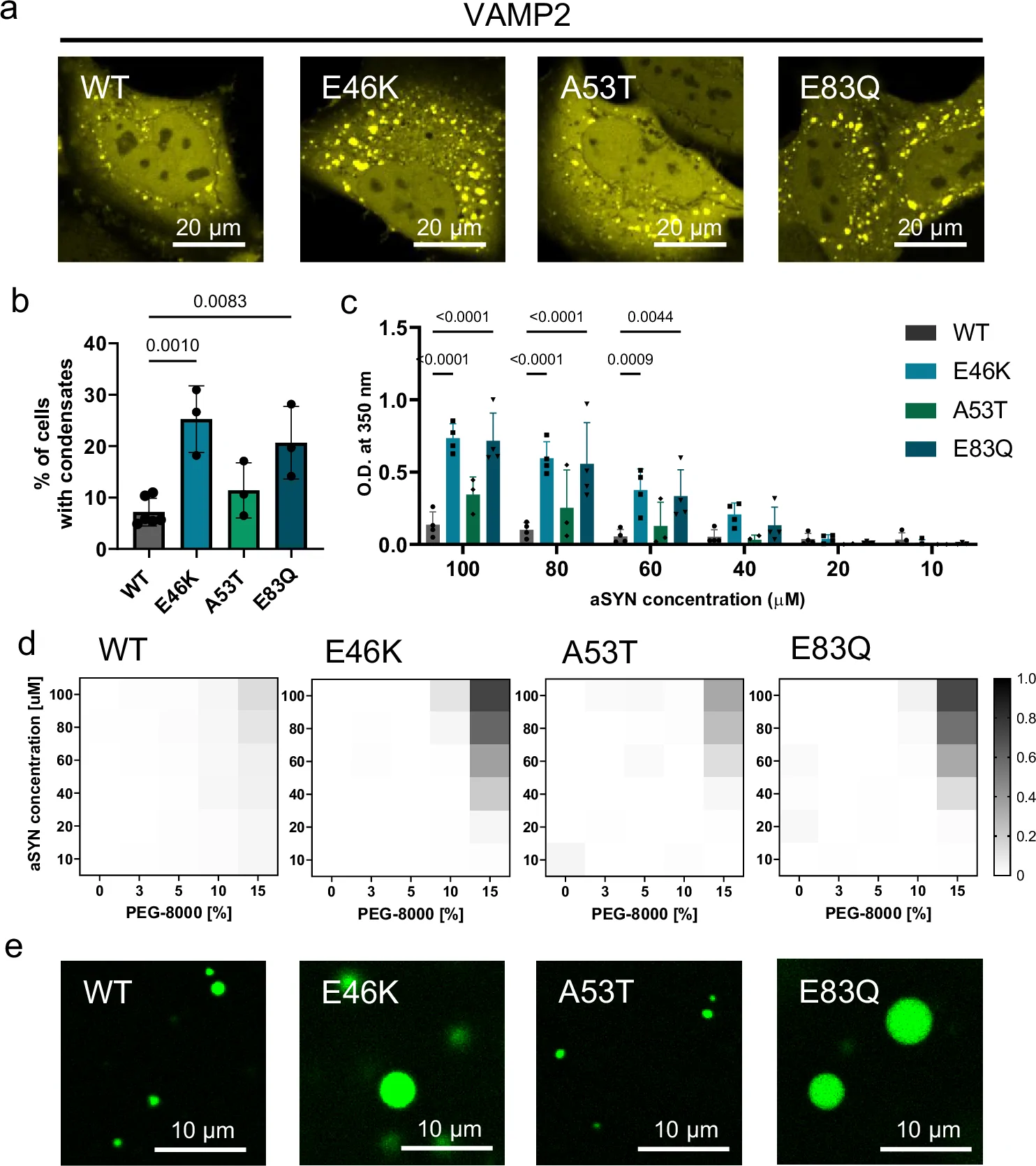
aSYN disease variants show increased condensate formation. **a** Condensate formation of aSYN-YFP variants upon co-expression with VAMP2 in HeLa cells. Increased condensate formation is observed for E46K-YFP and E83Q-YFP. Representative images from a minimum of 3 biological replicates. **b** Quantification of condensate formation in cells, data derived from IncuCyte screening with 16 images per well, three wells per biological replicate; n indicates biological replicates, with *n* = 6 for WT and *n* = 3 for E46K, A53T and E83Q, respectively. Data are shown as mean ± s.d. One-way ANOVA with Dunnett’s multiple comparisons test. **c** Quantification of turbidity measurements (O.D. at 350 nm) of aSYN phase separation in vitro; n indicates independent replicates from two separate batches of recombinant aSYN, with *n* = 4 for WT, E46K and E83Q and *n* = 3 for A53T. Data are shown as mean ± s.d. Two-way ANOVA with Dunnett’s multiple comparisons test. All significant *p* values are displayed on the graph. **d** Heat maps of turbidity measurements (O.D. at 350 nm) representing aSYN phase separation in vitro. Data represent 4 independent replicates for WT, E46K and E83Q and 3 independent replicates for A53T, from two separate batches of recombinant aSYN respectively. **e** Confocal images showing phase separation of aSYN variants in the presence of 2 mM Ca^2+^ and 15% PEG 8000, aSYN was used at 100 µM, supplemented with 1% aSYN-Alexa 488; 25 mM HEPES buffer pH 7.4. Representative images from 3 independent replicates for WT and E46K, and 2 independent replicates for A53T and E83Q.

Our previous work showed that aSYN phase separation is regulated by VAMP2^12^. Using this model of VAMP2-mediated aSYN phase separation in HeLa cells, we now investigate the phase separation behaviour of disease-associated aSYN variants. aSYN variants, C-terminally tagged with YFP, were co-transfected with VAMP2 in HeLa cells and imaged 24 h post transfection. We find that aSYN variants E46K, A53T, and the newly described DLB-associated variant, E83Q^17^ undergo VAMP2-mediated condensate formation (Fig. 1a). Condensate formation is enhanced for E46K and E83Q, while A53T shows similar levels compared to WT (Fig. 1b). Notably, similar patterns of phase separation are seen in vitro, where E46K and E83Q show increased droplet formation, while A53T is not significantly increased (Fig. 1c–e).

**Fig. 2:**
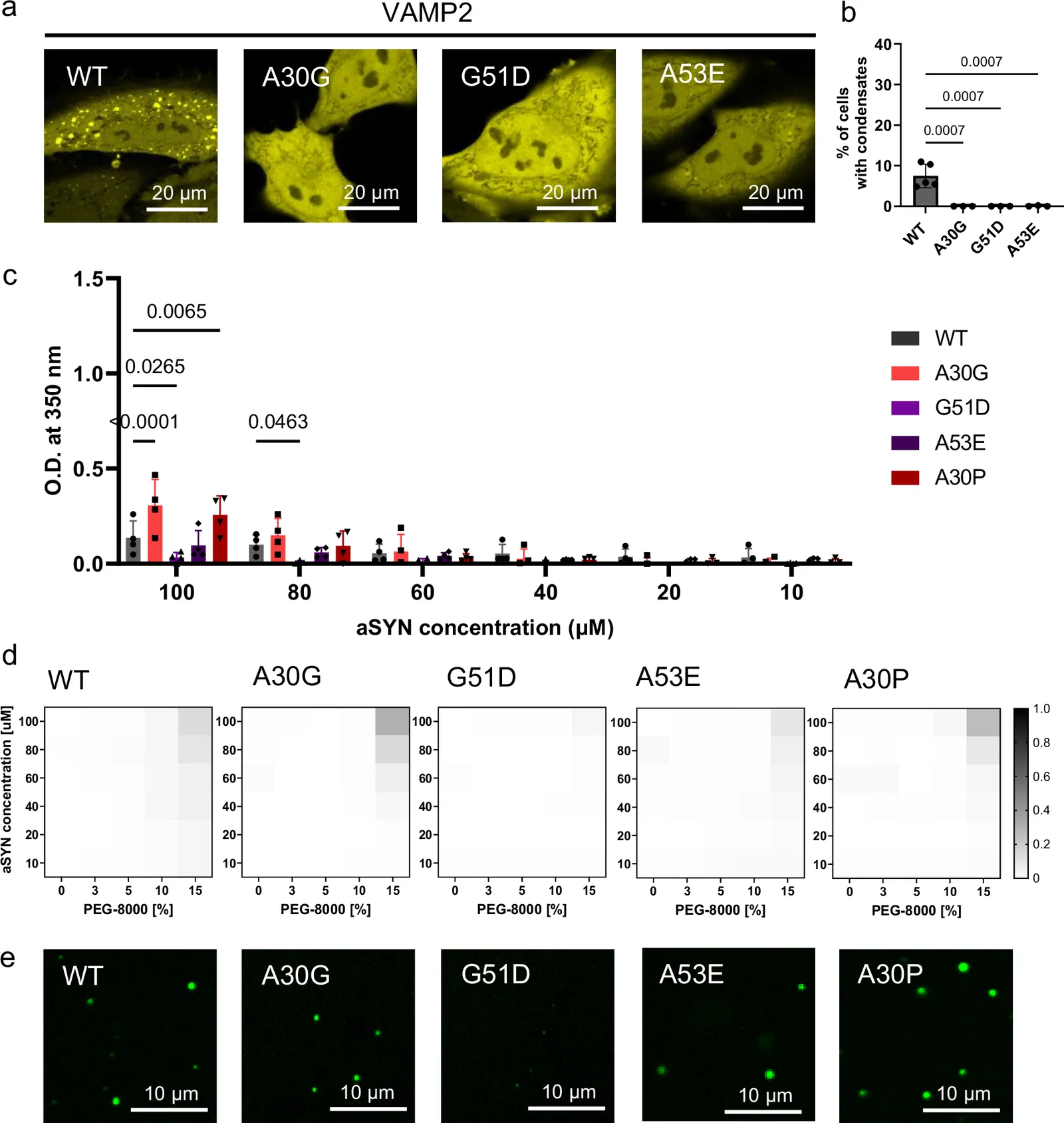
aSYN disease variants show decreased condensate formation. **a** Condensate formation of aSYN-YFP variants upon co-expression with VAMP2 in HeLa cells. No condensate formation is observed for A30G and G51D, only small condensates are seen in a subset of cells for A53E. Representative images from a minimum of 3 biological replicates. **b** Quantification of condensate formation in cells, data derived from IncuCyte screening with 16 images per well, three wells per biological replicate; n indicates biological replicates, with *n* = 5 for WT and *n* = 3 for A30G, G51D and A53E, respectively. Data are shown as mean ± s.d. One-way ANOVA with Dunnett’s multiple comparison test. **c** Quantification of turbidity measurements (O.D. at 350 nm) of aSYN phase separation in vitro. *n* indicates independent replicates from two separate batches of recombinant aSYN, with *n* = 4 for WT, A30G, G51D, A53E and A30P. Data are shown as mean ± s.d. Two-way ANOVA with Dunnett’s multiple comparisons test. All significant *p* values are displayed on the graph. **d** Heat maps of turbidity measurements (O.D. at 350 nm) representing aSYN phase separation in vitro. Data represent 4 independent replicates for WT, A30G, G51D, A53E and A30P, from two separate batches of recombinant aSYN respectively. **e** Confocal images showing phase separation of aSYN variants in vitro in the presence of 2 mM Ca^2+^ and 15% PEG 8000, aSYN was used at 100 µM, supplemented with 1% aSYN-Alexa 488; 25 mM HEPES buffer pH 7.4. Representative images from 3 independent replicates for WT and A53E, 2 independent replicates for A30G, G51D and A30P.

In our previous study, we reported that A30P fails to form condensates in cells^12^. Here we observe that other aSYN variants, including A30G and G51D also show no condensate formation in HeLa cells (Fig. 2a, b). Additionally, A53E has markedly reduced levels of condensate formation in HeLa cells, with a small subset of cells forming condensates (Fig. 2a). We next examined whether these variants would form condensates in vitro. All variants were capable of undergoing phase separation in vitro, albeit significantly less for G51D and slightly less for A53E, compared to WT (Fig. 2c–e). A30P and A30G however, showed higher phase separation compared to WT, in contrast to our observations in cells.

**Fig. 3:**
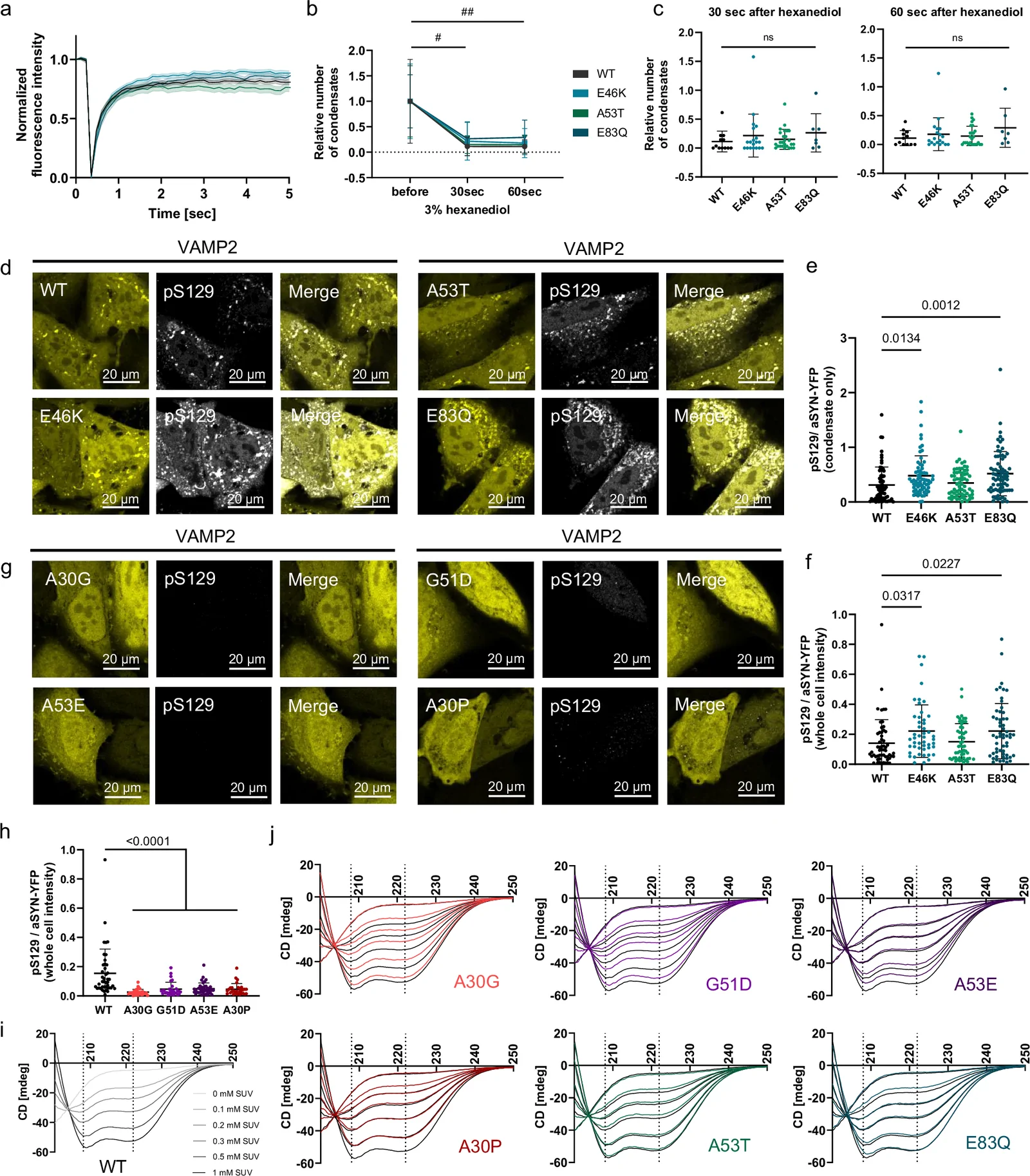
Condensate characterization, aSYN phosphorylation and lipid binding. **a** Fluorescence recovery after photobleaching (FRAP) experiments of aSYN-YFP condensates in HeLa cells demonstrating rapid aSYN recovery for all variants. *n* indicates number of condensates measured from a minimum of 4 biological replicates, with *n* = 61, 36, 41 and 31 condensates for WT, E46K, A53T and E83Q, respectively. Data are shown as mean ± s.e.m. **b** Quantification of aSYN condensate dispersal in HeLa cells upon incubation with 3% 1,6-hexanediol showing a decrease in the relative number of condensates per cell. n indicates number of cells from a minimum of 3 biological replicates, with *n* = 12, 19, 26, 7 cells for WT, E46K, A53T and E83Q, respectively. Data are shown as mean ± s.d. RM Two-way ANOVA with Geisser-Greenhouse correction and Dunnett’s multiple comparisons test. # all variants showed a significant decrease in number of condensates after 30 sec. *p* = 0.0013 for WT, *p* < 0.0001 for E46K and A53T, and *p* = 0.0013 for E83Q. ## all variants showed a significant decrease in number of condensates after 60 sec. *p* = 0.0026 for WT, *p* < 0.0001 for E46K and A53T, and *p* = 0.0010 for E83Q. **c** Comparison of number of aSYN condensates in cells after 30 sec and 60 sec treatment with 1,6 hexanediol. n indicates number of cells from a minimum of 3 biological replicates, with *n* = 12, 19, 26 and 7 cells for WT, E46K, A53T and E83Q, respectively. Data are shown as mean ± s.d. One-way ANOVA with Dunnett’s multiple comparisons test. **d** Immunocytochemistry for aSYN Serine 129 phosphorylation (pS129) in HeLa cells co-expressing aSYN-YFP variants with VAMP2. pS129 immunoreactivity is seen in condensates and within the cytosol, with increased pS129 reactivity for E46K and E83Q. Representative images from 5 biological replicates. **e** Quantification of pS129 immunoreactivity within condensates. *n* indicates number of cells analysed from 5 biological replicates with *n* = 68, 67, 63 and 74 cells for WT, E46K, A53T and E83Q, respectively. Data are shown as mean ± s.d. One-way ANOVA with Dunnett’s multiple comparisons test. **f** Quantification of whole cell pS129 immunoreactivity normalized to total aSYN-YFP intensity. *n* indicates number of cells from 4 biological replicates with *n* = 56, 48, 53 and 57 cells for WT, E46K, A53T and E83Q, respectively. Data are shown as mean ± s.d. One-way ANOVA with Dunnett’s multiple comparisons test. **g** Immunocytochemistry for aSYN Serine 129 phosphorylation (pS129) in HeLa cells co-expressing non-condensate forming aSYN-YFP variants with VAMP2. A30G, G51D, A53E and A30P show weak pS129 immunoreactivity. Representative images from 3 biological replicates. **h** Quantification of whole cell pS129 immunoreactivity normalized to total aSYN-YFP intensity. *n* indicates number of cells from 3 biological replicates with *n* = 42, 32, 29, 38 and 30 cells from WT, A30G, G51D, A53E and A30P, respectively. Data are shown as mean ± s.d. One-way ANOVA with Dunnett’s multiple comparisons test. *p* < 0.0001 for WT vs A30G, G51D, A53E and A30P, respectively. **i** Circular dichroism (CD) spectra for aSYN WT without lipids and in the presence of increasing concentrations of small unilamellar vesicles (SUVs), showing transition from random coil conformation to increasing alpha-helical content. Data represent 8 independent replicates. All SUVs constitute 100% POPS. **j** Direct comparison of CD spectra of different aSYN variants compared to WT, showing decreased alpha-helical content for A30G, G51D, A53E and A30P. A30P shows the highest reduction in alpha-helical content compared to other aSYN variants. A53T and E83Q show no difference to WT. All SUVs constitute 100% POPS. Data represent 4 independent replicates for A30G, G51D, A53E, A53T, E83Q, and 6 independent replicates for A30P, from two batches of recombinant aSYN.

Phase separation can be affected by addition of fluorescent tags or labels^18,19^; therefore, we performed additional experiments evaluating the impact of respective tags on aSYN condensates (Supplementary Figs. 1 and 2). aSYN condensates can be seen in HeLa cells when YFP is replaced with mScarlet, wherein WT-mScarlet and E46K-mScarlet form condensates to a similar extent to YFP tagged proteins (Supplementary Fig. 1a, b). Additionally, aSYN condensate formation is also evident when untagged aSYN is co-transfected with VAMP2 in HeLa cells (Supplementary Fig. 1c). Similarly, in vitro experiments show comparable phase separation propensity for labelled and non-labelled protein (Supplementary Fig. 2), for both WT and E46K. In addition, we show that whole cell and cytosolic aSYN levels are comparable between aSYN variants, confirming that divergent patterns of condensate formation in cells are not caused by differences in expression levels (Supplementary Fig. 3a). Finally, to determine whether non-phase separating aSYN variants would form condensates at a later time point, we examined condensate formation at 48 h post transfection. We find that variants which do not form condensates at 24 h; A30G, G51D, A53E and A30P, also do not form condensates after 48 h (Supplementary Fig. 3b).

### Condensate characterization, aSYN phosphorylation and lipid binding

We next probed aSYN condensate properties using fluorescence recovery after photobleaching (FRAP) and 1,6 hexanediol-induced dispersion experiments at 24 h post transfection. FRAP analysis showed that aSYN condensates, regardless of the variant, exhibit rapid recovery kinetics, indicating a high degree of mobility between the condensates and the cytosolic aSYN pool for all variants (Fig. 3a), with no significant differences in half-life and mobile fraction (Supplementary Fig. 4a). Further to this, we also measured FRAP kinetics at a longer time point, 48 hours post transfection, which showed similar fluorescence recovery profiles with no statistically significant differences (Supplementary Fig. 4b, c). We next tested whether small aliphatic alcohols, such as 1,6-hexanediol, lead to dispersion of aSYN condensates in cells^20^. Again, all aSYN condensates, regardless of the aSYN variant, dispersed upon treatment with 1,6-hexanediol (Fig. 3b) with no significant differences between variants (Fig. 3c).

Since phosphorylation of aSYN at Serine 129 is a well-established pathological marker in synucleinopathies, with increased levels seen in Lewy bodies^21,22^ and in response to fibril-induced aggregation in neurons^23^, we next determined the phosphorylation status of aSYN at Serine 129 (pS129) in condensates. Condensates were consistently pS129 positive, with a significant increase observed in the high-condensate-forming variants, E46K and E83Q (Fig. 3d, e). Interestingly, A53T, which is highly aggregation prone^24–26^, exhibits comparable pS129 staining to WT. We next analyzed whole cell pS129 staining, which correspondingly also showed significantly higher levels for E46K and E83Q (Fig. 3f). In addition, aSYN variants which do not form condensates have significant lower pS129 levels compared to WT (Fig. 3g, h).

Next, we hypothesized that lipid binding influences aSYN condensate formation, since A30P, being one of the variants which does not form condensates in cells, is well known to have reduced lipid binding properties^27^. We compared lipid binding of aSYN variants using circular dichroism experiments where aSYN shows a transition from random coil to alpha-helix in the presence of increasing amounts of small unilamellar vesicles (SUVs) (Fig. 3i). A30P shows reduced alpha-helical content in the presence of SUVs, consistent with impaired lipid binding. To a lesser degree, A30G, G51D, and A53E also show reduced alpha-helical content (Fig. 3j), while A53T and E83Q show comparable alpha-helical content to WT. Together, these results suggest that, for A30P and A30G, an absence of condensate formation in cells is likely a result of reduced lipid binding, but not their intrinsic phase separation propensity, as they show higher condensate formation compared to WT in vitro. For G51D and A53E, lack of condensate formation in cells could result from a combination of both factors, lower intrinsic phase separation propensity, but also lipid binding deficiency.

### Lipid binding promotes aSYN phase separation

**Fig. 4:**
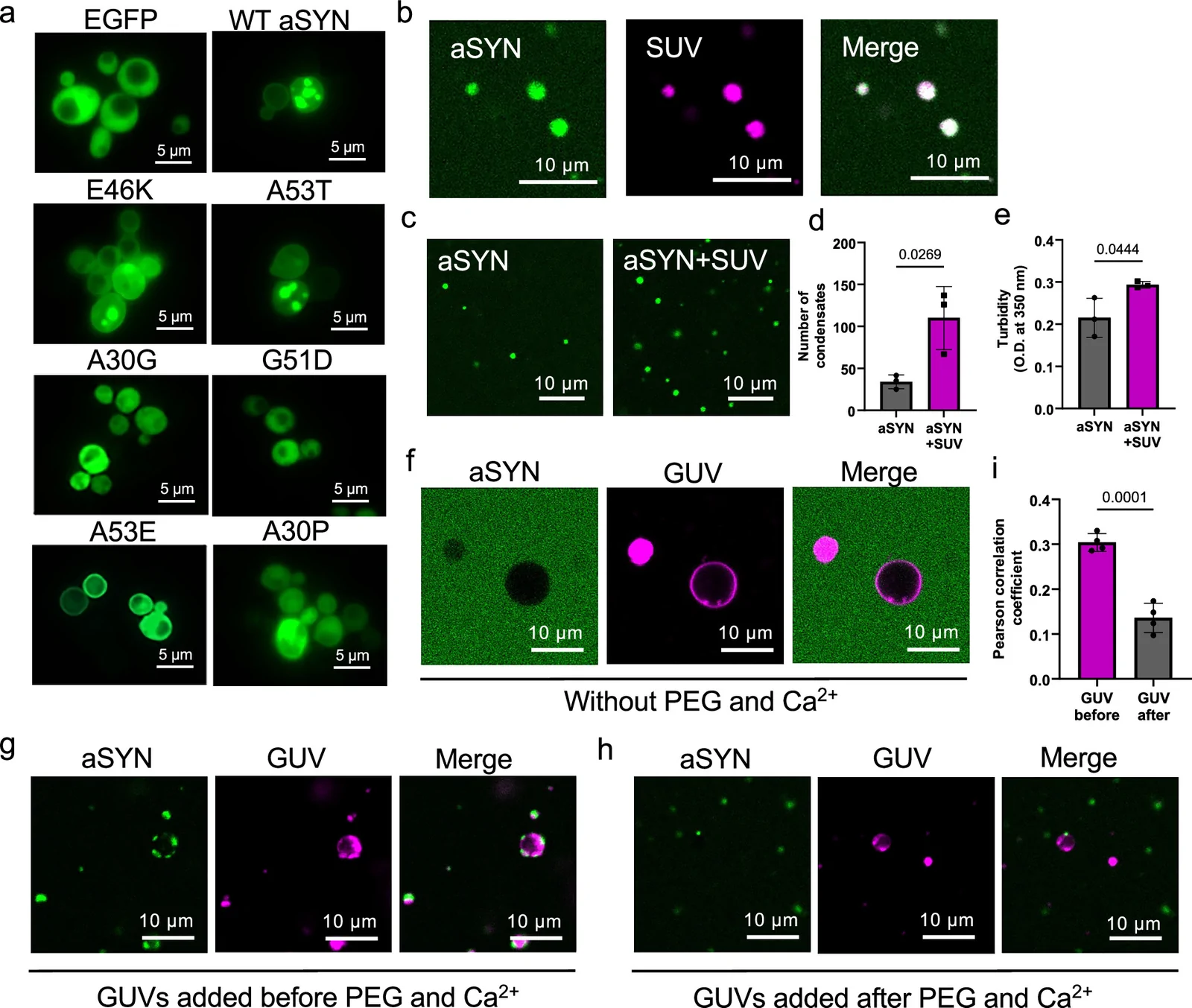
aSYN inclusion formation in yeast and role of lipid membranes. **a** aSYN-EGFP inclusion formation in yeast showing EGFP as a control with cytosolic distribution. aSYN WT-EGFP, E46K-EGFP and A53T-EGFP form inclusions. A30G-EGFP, G51D-EGFP, and A30P-EGFP show cytosolic distribution, while A53E-EGFP shows cytosolic distribution and plasma membrane enrichment. Representative images from 3 biological replicates. **b** aSYN phase separation in vitro in the presence of 1 mM SUVs, 2 mM Ca^2+^ and 15% PEG 8000. aSYN was used at 80 µM, supplemented with 1% aSYN-Alexa 488; 25 mM HEPES buffer pH 7.4. SUVs containing 60% DOPC, 30% DOPS, and 10% cholesterol, labelled with 0.1% PE-Cy5. Representative images from 3 independent replicates. **c** aSYN phase separation with 2 mM Ca^2+^ and 15% PEG 8000, without and with 1 mM SUVs; 25 mM HEPES buffer pH 7.4. Representative images from 3 independent replicates. **d** Quantification of number of condensates from confocal images in (**c**). n indicates 3 independent replicates, with a total of 17 images from aSYN alone and 16 images from aSYN + SUV. Data are shown as mean ± s.d. Unpaired two-tailed *t* test. **e** Quantification of turbidity measurements (O.D. at 350 nm) of aSYN phase separation in vitro with 2 mM Ca^2^ and 15% PEG 8000 without and with 1 mM SUVs; 25 mM HEPES buffer pH 7.4. Turbidity of control samples with SUVs but omitting aSYN were subtracted from aSYN + SUV condition. *n* indicates 3 independent replicates. Data are shown as mean ± s.d. Unpaired two-tailed *t* test. **f** aSYN incubated with 1 mM GUVs. aSYN was used at 100 µM, supplemented with 1% aSYN-Alexa 488; 25 mM HEPES buffer pH 7.4. GUVs containing 60% DOPC, 30% DOPS, and 10% cholesterol, labelled with 0.1% PE-Cy5. Representative images from 2 independent replicates. **g**, **h** aSYN incubated in the presence of 2 mM Ca^2+^ and 15% PEG 8000 to induce phase separation. 1 mM GUVs were added either before (**g**) or after aSYN phase separation was induced (**h**). aSYN was used at 100 µM, supplemented with 1% aSYN-Alexa 488; 25 mM HEPES buffer pH 7.4. Representative images from 4 independent replicates. **i** Pearson correlation analysis of aSYN and GUV co-localisation of data in (**g**, **h**). *n* indicates 4 independent replicates, with a total of 23 and 28 images analyzed from ‘GUV before’ and ‘GUV after’ condition, respectively. Data are shown as mean ± s.d. Unpaired two-tailed *t* test.

Our results so far show that aSYN variants can have contrasting effects on VAMP2-mediated condensate formation in cells; where some variants facilitate but others abolish condensate formation. To further confirm that the observed phenotypes for the different variants translate to other model systems, we expressed aSYN in budding yeast, which has been established as a model for a distinct cellular environment in which aSYN displays membrane enrichment and inclusion formation^28^. Strikingly, we find that the pattern of inclusion formation of aSYN variants reflects the phenotypes observed upon VAMP2-mediated aSYN condensate formation in HeLa cells. While WT, E46K and A53T form inclusions in yeast, A30P, A30G, G51D and A53E show no inclusion formation (Fig. 4a). Some cells expressing A53E, however, show plasma membrane localization, which may demonstrate a pre-inclusion state and might relate to its intermediate condensate phenotype in HeLa cells.

Due to the potential role of lipid membranes on aSYN condensate formation, we next tested whether lipid membranes can affect aSYN phase separation in vitro. We first confirmed that aSYN condensates incorporate SUVs (Fig. 4b), consistent with our previous finding that aSYN condensates in cells cluster vesicles^12^. We further find that SUVs can increase aSYN droplet formation in vitro (Fig. 4c, d), which is also seen in quantitative turbidity measurements (Fig. 4e). Since SUVs are too small to be imaged individually, we turned to giant unilamellar vesicles (GUVs) as another model system. When aSYN, labelled with Alexa 488, was incubated with GUVs, aSYN was uniformly distributed outside of GUVs, with no visible enrichment on the surface (Fig. 4f). However, when condensate formation was subsequently induced by addition of Ca^2+^ and PEG to the aSYN-GUV mixture, we see aSYN condensates form on the GUV surface (Fig. 4g). If GUVs were added after induction of phase separation with Ca^2+^ and PEG, aSYN droplets mostly remained in solution, showing significantly less co-localization with GUVs (Fig. 4h, i). This suggests that aSYN condensates form on GUVs rather than simply binding to their surface, supporting the concept that aSYN condensates can nucleate on lipid membranes.

**Fig. 5:**
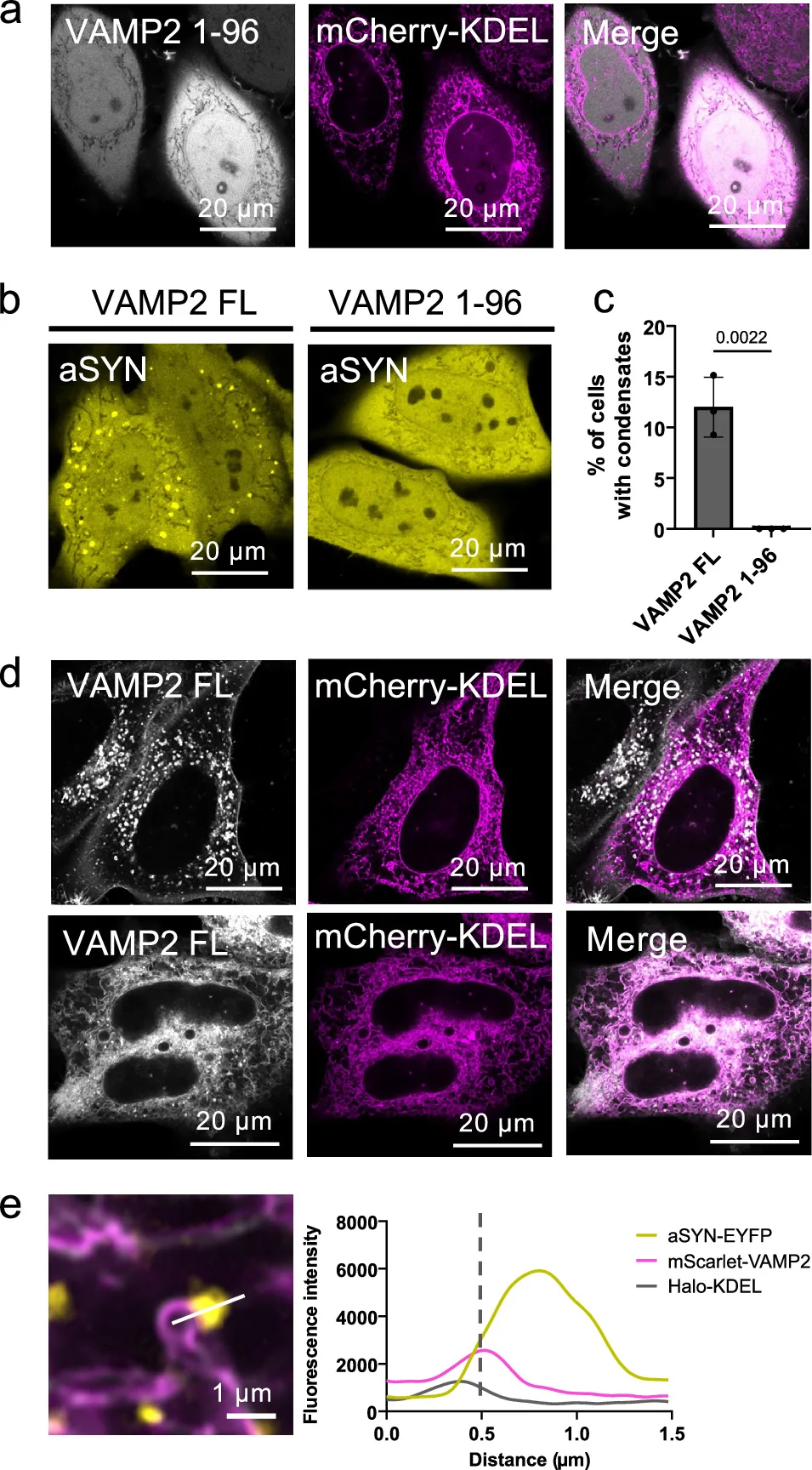
aSYN condensate formation on lipid membranes in cells. **a** HeLa cells co-expressing ER marker mCherry-KDEL and mTurquoise-VAMP2 1-96, showing cytosolic distribution of VAMP2 1-96 when its transmembrane domain is deleted. Representative images from 3 biological replicates. **b** HeLa cells co-expressing WT aSYN-YFP with full-length VAMP2 (FL) show aSYN condensate formation, while cells co-expressing WT aSYN-YFP with VAMP2 1-96 show no condensate formation. Representative images from 3 biological replicates. **c** Quantification of condensate formation in cells expressing WT aSYN-YFP with either VAMP2 FL or VAMP2 1-96, data derived from IncuCyte screening with 16 images per well, three wells per biological replicate; n indicates 3 biological replicates. Data are shown as mean ± s.d. Unpaired two-tailed *t* test. **d** HeLa cells expressing mTurquoise-VAMP2 FL show mixed localization with both vesicular and ER distribution, mCherry-KDEL is used to visualize ER network. Representative images from 3 biological replicates. **e** HeLa cells co-expressing aSYN-YFP, Halo-KDEL (ER marker), and mScarlet-VAMP2 FL show aSYN condensates on VAMP2 positive ER membranes. See Supplementary Fig. 5 for separate colour images of each marker and respective overlays. Representative images from 3 biological replicates.

We next returned to our HeLa cell model to determine whether lipid membranes are necessary for VAMP2-mediated aSYN condensate formation in cells. To this end, we expressed a truncated variant of VAMP2 (VAMP2 1-96), which lacks the C-terminal transmembrane domain. VAMP2 1-96 localized to the cytosol (Fig. 5a), and, when co-expressed with aSYN-YFP, was not able to induce aSYN condensate formation (Fig. 5b, c). Full-length VAMP2 localizes to vesicular structures and ER membranes upon overexpression in HeLa cells (Fig. 5d). We therefore investigated the co-localisation of aSYN condensates with intracellular organelles (Supplementary Fig. 5a). We find that aSYN condensates reside on the ER tubule network, consistent with the enrichment of VAMP2 on ER membranes in our model system. Time resolved imaging shows that aSYN condensates move on the ER network showing fluid-like properties (Supplementary videos 1, 2). Furthermore, using three-color, live-cell, high-resolution Airyscan imaging, we confirm that aSYN condensates are associated with VAMP2-positive ER membranes (Fig. 5e and Supplementary Fig. 5b). Together, these findings support the concept that aSYN lipid binding is necessary for its condensate formation in cells.

### Synaptic enrichment of aSYN disease variants

**Fig. 6:**
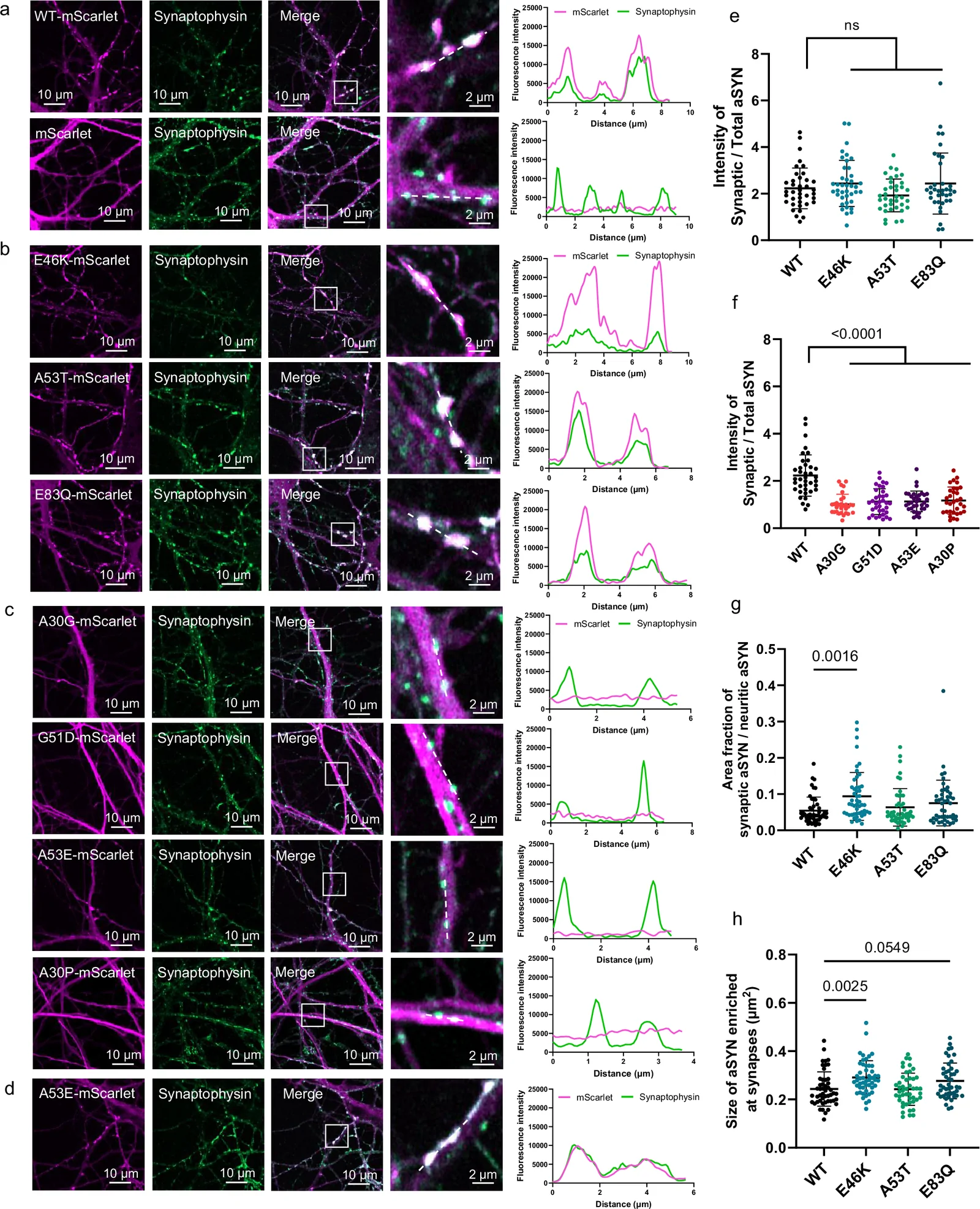
Synaptic enrichment of aSYN disease variants. **a** WT aSYN-mScarlet and mScarlet expressed in SNCA-knock out iPSC-derived cortical neurons. Synaptophysin-mEmerald was expressed as synapse marker. Zoomed in images and line diagrams for synaptic enrichment are shown. Representative images from 4 biological replicates. **b** E46K-, A53T- and E83Q-mScarlet expressed in SNCA-knock out iPSC-derived cortical neurons, showing synaptic enrichment. Representative images from 4 biological replicates. **c** A30G-, G51D-, A53E- and A30P-mScarlet expressed in SNCA-knock out iPSC-derived cortical neurons, showing lack of synaptic enrichment. Representative images from 4 biological replicates. **d** Example images of A53E-mScarlet showing synaptic enrichment in certain terminals. **e** Quantification of aSYN synaptic enrichment displayed as ratio of intensity of aSYN at the synapse vs. total aSYN for WT, E46K, A53T and E83Q. Synaptic terminals were selected using the synaptophysin mask. n indicates number of fields of view from 4 biological replicates with n = 38, 40, 37 and 36 fields of view for WT, E46K, A53T and E83Q, respectively. Data shown as mean ± s.d. One-way ANOVA with Dunnett’s multiple comparison test. **f** Quantification of aSYN synaptic enrichment displayed as ratio of intensity of aSYN at the synapse vs. total aSYN. Synaptic terminals were selected using the synaptophysin mask. n indicates number of fields of view from 4 biological replicates with *n* = 38, 30, 35, 34, 32 fields of view for WT, A30G, G51D, A53E and A30P, respectively. Data shown as mean ± s.d. One-way ANOVA with Dunnett’s multiple comparison test. *p* < 0.0001 for WT vs A30G, G51D, A53E and A30P, respectively. **g** Quantification of area fraction of aSYN-enriched synapses relative to neurite area excluding cell bodies. n indicates number of fields of view from 5 biological replicates with *n* = 49, 50, 47, 46 fields of view for WT, E46K, A53T and E83Q, respectively. Data shown as mean ± s.d. One-way ANOVA with Dunnett’s multiple comparison test. **h** Quantification of the size of aSYN-enriched synapses. *n* indicates number of fields of view from 5 biological replicates with *n* = 49, 50, 47 and 46 fields of view for WT, E46K, A53T and E83Q, respectively. Data are shown as mean ± s.d. One-way ANOVA with Dunnett’s multiple comparison test.

To examine whether aSYN variants may have different patterns of cellular localization in neurons, we utilized an iPSC-derived neuron model to determine levels of synaptic enrichment of aSYN. CRISPR-Cas9 gene editing was used to knock out endogenous *SNCA* in an iPSC line with stable NGN2 overexpression, followed by differentiation to cortical neurons^29,30^ (for characterization of SNCA-KO iPSCs, see Supplementary Fig. 6). Lentiviruses were used to transduce neurons with WT aSYN or aSYN disease-variants tagged to the fluorescent protein mScarlet to enable live cell imaging. WT aSYN-mScarlet shows enriched localization at the synapse, although a cytosolic fraction is also present (Fig. 6a). mScarlet on its own shows no synaptic enrichment with an evenly cytosolic distribution (Fig. 6a). We proceeded to investigate the distribution of the aSYN disease variants in neurons. E46K, A53T and E83Q variants show synaptic enrichment, similar to WT (Fig. 6b). However, variants that do not undergo VAMP2-mediated phase separation in HeLa cells, fail to enrich at synaptic terminals in neurons. This is the case for A30G, G51D and A30P, which have an evenly distributed cytosolic expression, similar to mScarlet alone (Fig. 6c). A53E also exhibits a predominant cytosolic localization; however, we observe synaptic localization in a subset of terminals (Fig. 6c, d). We quantified the intensity ratio of aSYN at synapses compared to total aSYN levels using a synaptophysin mask. This confirms synaptic enrichment of WT, E46K, A53T and E83Q, while for A30G, G51D, A53E and A30P synaptic localization is significantly reduced (Fig. 6e/f). We further quantified the area fraction of synaptic aSYN to aSYN in neurites, as well as the size of aSYN enriched synapses. We find that the area fraction is significantly increased for E46K (Fig. 6g), and that the size of aSYN enriched synapses is significantly increased for both E46K and E83Q (Fig. 6h). No increase in the size of aSYN-enriched synapses is seen for A53T, in line with our in vitro and HeLa cell observations where A53T behaves similarly to WT. To confirm that changes in synaptic enrichment are not caused due to differential protein expression of the respective variants, we performed western blotting for aSYN-mScarlet, showing no significant differences between expression levels among the aSYN variants (Supplementary Fig. 7). This suggests that differential expression is not the cause of increased or decreased synaptic localization.

**Fig. 7:**
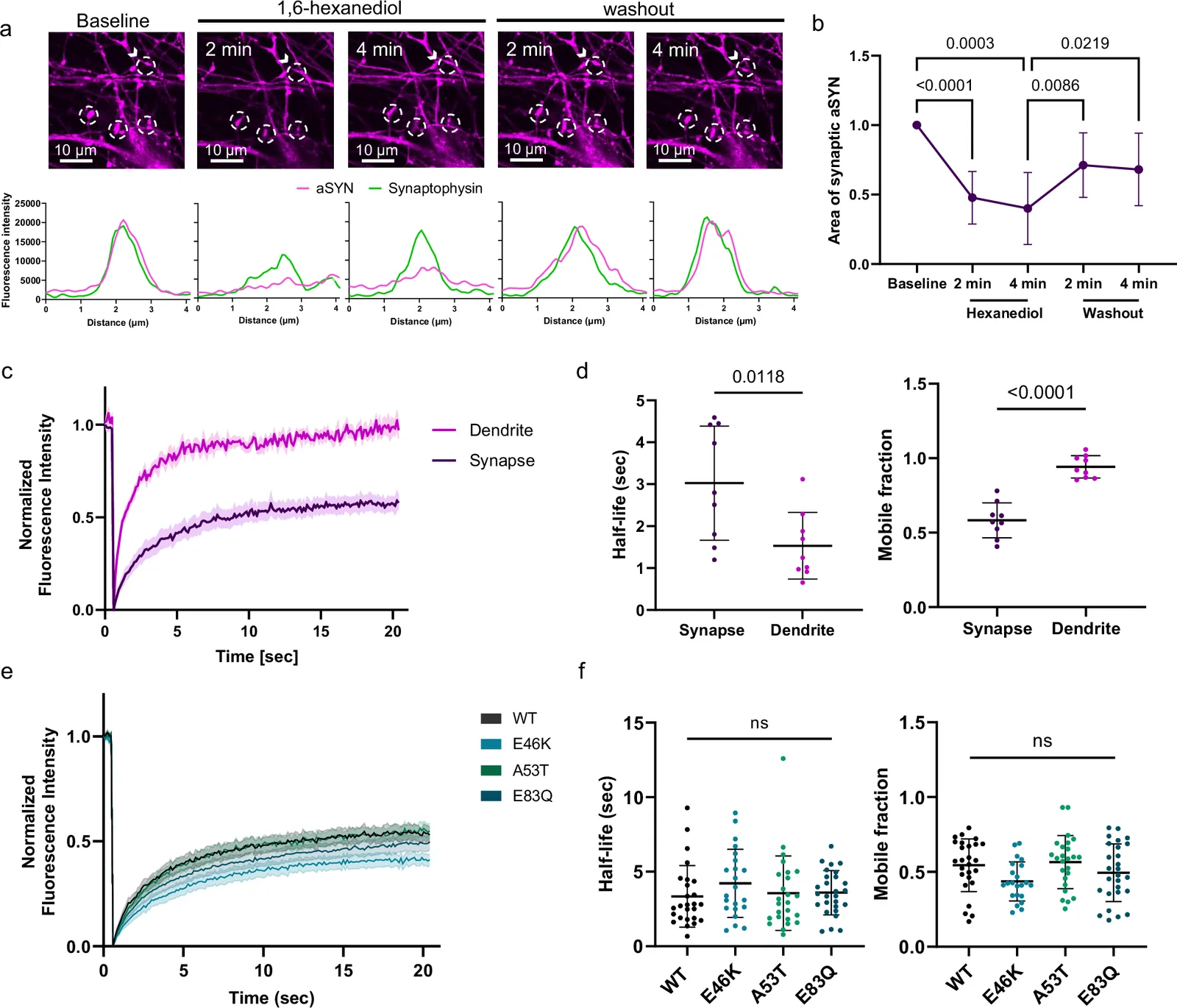
Characterization of aSYN enriched at the synapse. **a** WT aSYN-mScarlet expressed in SNCA-knock out iPSC-derived cortical neurons showing synaptic enrichment at baseline. Neurons incubated with 3% 1,6-hexanediol were imaged at 2 and 4 min, then 1,6-hexanediol was washed out and new media added. Synaptophysin-mEmerald was expressed as synapse marker. Representative images from 4 biological replicates. **b** Quantification of aSYN synaptic enrichment at baseline, upon incubation with 3% 1,6-hexanediol and upon washout. *n* indicates number of fields of view analyzed, with *n* = 10 fields of view from 4 biological replicates. Data are shown as mean ± s.d. RM One-way ANOVA with Geisser-Greenhouse correction and Tukey’s multiple comparisons test. Significant *p* values are: *p* < 0.0001 for baseline vs hexanediol 2 min, *p* = 0.0003 for baseline vs hexanediol 4 min, *p* = 0.0226 for baseline vs washout 2 min, *p* = 0.0238 for baseline vs washout 4 min. *p* = 0.0452 for hexanediol 2 min vs washout 2 min, *p* = 0.0086 for hexanediol 4 min vs washout 2 min, and *p* = 0.0219 for hexanediol 4 min vs washout 4 min. **c** Fluorescence recovery after photobleaching (FRAP) for aSYN-mScarlet in dendrites and at synapses showing faster and greater extent of recovery in dendrites compared to synapses. *n* indicates number of individual FRAP measurements from 3 biological replicates, with *n* = 9 for synapses and dendrites, respectively. Data are shown as mean ± s.e.m. **d** Quantification of half-life and mobile fraction of fluorescence recovery kinetics after curve fitting with a one phase decay equation. *n* indicates number of FRAP measurements from 3 biological replicates, with *n* = 9 for synapses and dendrites, respectively. Data are shown as mean ± s.d. Unpaired two-tailed t test. **e** FRAP for WT-, E46K-, A53T- and E83Q-mScarlet at synapses. n indicates number of FRAP measurements from 3 biological replicates with *n* = 26, 22, 25 and 27 for WT, E46K, A53T, and E83Q, respectively. Data are shown as mean ± s.e.m. **f** Quantification of half-life and mobile fraction of FRAP kinetics after curve fitting with a one phase decay equation. n indicates number of FRAP measurements from 3 biological replicates, with *n* = 26, 22, 25 and 27 for WT, E46K, A53T and E83Q, respectively. Data are shown as mean ± s.d. One-way ANOVA with Dunnett’s multiple comparisons test.

As has been shown for other soluble phase separating proteins, such as dynamin^31^ and endophilin A1^32^, we tested whether synaptic enrichment of aSYN is sensitive to 1,6-hexanediol. We see that aSYN rapidly disperses from synapses upon incubation with 1,6-hexanediol. Furthermore, we observe a recovery of synaptic aSYN enrichment upon 1,6-hexanediol washout (Fig. 7a, b). To characterise the behaviour of aSYN at synapses further, we performed FRAP experiments comparing the mobility of aSYN at the synapse and in dendrites. We observe a distinct recovery profile for synaptic aSYN (Fig. 7c), showing less recovery in synapses compared to dendrites, reflected in a significantly higher half-life and lower mobile fraction (Fig. 7d), implying that aSYN is actively retained in synapses and not freely diffusive. We subsequently performed FRAP experiments at the synapse for all synaptically enriched aSYN variants. We find that all aSYN variants retain high mobility at the synapse, consistent with the fluorescence recovery profiles seen for aSYN condensates in HeLa cells. E46K shows a small decrease in the extent of recovery (Fig. 7e), however no significant difference is seen for recovery half-life or the mobile fraction (Fig. 7f). The link between condensate formation of aSYN disease variants in HeLa cells and their synaptic enrichment in neurons suggests that phase separation might be important to regulate aSYN subcellular compartmentalisation in neurons. Taken together, our findings demonstrate that while aSYN condensate formation is increased for some disease variants such as E46K and E83Q, this is not the case for other variants, including A30G, G51D, A53E and A30P, indicating that impaired condensate formation, increased or decreased, may contribute to disease in distinct ways.

## Discussion

In summary, we find that disease associated aSYN variants exhibit a dichotomy in their ability to undergo biomolecular condensation, wherein only E46K and E83Q demonstrate enhanced condensate formation both in cells and in vitro. In contrast, A30P, A30G, G51D and A53E form no or very few condensates in cells. Our findings related to E46K are consistent with previously published work which has demonstrated that E46K has increased phase separation propensity in vitro^10,33^. Enhanced phase separation of E83Q, a variant associated with dementia with Lewy bodies^17,34^, has not been described before. Interestingly, A53T, the most widely studied aSYN disease variant which is directly associated with increased aggregation^24,25,35^, displays phase separation comparable to WT^10,33^.

We next performed FRAP experiments on condensates in HeLa cells, showing fast fluorescence recovery for all aSYN variants. In line with this, we also observe fast dispersion of condensates upon incubation with 1,6 hexanediol for all aSYN variants. Collectively, our data therefore suggest that in our HeLa cell model, under the specific conditions and timelines used, VAMP2-mediated aSYN condensates, irrespective of the disease-associated mutations, are dynamic structures lacking significant aggregation into insoluble states. In addition, we show that aSYN condensates exhibit increased pS129 immunoreactivity, with E46K and E83Q variants showing greater levels of pS129, while A53T is comparable to WT. Recent studies have called into question the strict association between pS129 and pathological aSYN aggregation, suggesting that pS129 is dynamically regulated during neuronal activity^36,37^. Additionally, it was found that pS129 levels may be regulated by membrane binding, as aSYN bound to liposomes was a better substrate for PLK2-dependant S129 phosphorylation in vitro^37^. It was further found that aSYN disease variants, upon overexpression in neurons under non-aggregating conditions, exhibited different tendencies to undergo pS129 phosphorylation^38^. A53T showed similar pS129 levels compared to WT, while E46K showed significantly higher pS129. In contrast, aSYN variants with decreased membrane enrichment, A30P and G51D, had lower pS129 levels^38^. This pattern is congruent with our results in HeLa cells and could explain differences in pS129 levels in the absence of significant aggregation.

Four aSYN variants, A30P, A30G, G51D and A53E show absent or markedly reduced condensate formation in cells. A30P and A30G, however, undergo significant phase separation in vitro. This is consistent with previous work, showing that A30P undergoes condensate formation comparable to WT in solution^33,39^ but has not been reported for A30G before. Our circular dichroism experiments indicate reduced alpha-helicity of A30P in the presence of lipid vesicles, suggesting reduced lipid binding, consistent with previous studies^27,40,41^. Similarly, but to a lesser extent, A30G also shows reduced alpha-helicity in the presence of lipid vesicles. Therefore, the discrepancy between in vitro and cellular condensate formation by A30P and A30G is likely to be explained by their decreased membrane binding characteristics. We therefore tested whether lipid binding can facilitate aSYN phase separation. We show that lipid membranes (SUVs and GUVs) indeed foster aSYN droplet formation in vitro, and also that aSYN condensates in cells are localised to lipid membrane surfaces. Importantly, membrane associated phase separation has been described for other proteins, such as RIM and RIM-BP^42^ in association with voltage gated calcium channels and for Whi3^43^ and AGO^44^ proteins on GUVs and in association with the ER, supporting our observations of membrane surfaces acting as a scaffold for phase separation in line with recent views on the interplay between biomolecular condensates and lipid membranes and phase separation on two-dimensional membrane interfaces^45^. For G51D and A53E, we see reduced alpha-helicity in CD experiments, consistent with decreased lipid binding that has been described before^46–48^, however these variants additionally show lower droplet formation in vitro. Therefore, the lack of biomolecular condensate formation by these variants in cells could be due to a combined effect of lower intrinsic phase separation propensity and decreased membrane binding.

Finally, we show that the phenotype of VAMP2-mediated aSYN condensate formation in HeLa cells translates to the propensity of aSYN to enrich at synaptic terminals in neurons, with WT, E46K, A53T and E83Q showing synapse enrichment, while A30P, A30G, G51D and A53E exhibiting a predominant cytosolic distribution. A53E shows enrichment in some synaptic terminals, similar to our observations that A53E can form condensates in a subset of HeLa cells. Given that aSYN phase separation is influenced by VAMP2, and the ability of aSYN condensates to cluster vesicles in cells^12^, similar to Synapsin 1 condensation^49^, we propose that synaptic enrichment of aSYN could reflect condensate formation at the synapse. This is further evidenced by sensitivity of synaptic aSYN to 1,6-hexanediol, and differential FRAP kinetics. Lack of synaptic enrichment has been previously described for A30P^50^ and, more recently also for G51D^51^; however, this has not been shown for A30G and A53E, emphasizing that a depletion of aSYN from the synapse may be relevant to disease. With this, we suggest that in addition to aggregation, differential condensate formation by aSYN disease variants may have distinct cellular effects, either via protein redistribution from the synapse to other cellular compartments or via a direct effect on synaptic function, wherein aSYN may lose its ability to participate in vesicle clustering, exocytosis or endocytosis^9^. Similarly, enhanced phase separation may directly affect synaptic activity-dependent condensation and dispersion of aSYN at the synapse, causing changes in synaptic function, prior to aggregation. Previous studies on aSYN phase separation have linked subsequent liquid-to-solid transition of aSYN condensates in vitro to the formation of insoluble fibrils^10,15,16,52^. While this pathway may still hold true for variants that promote phase separation, we challenge this view as the only mechanism of aSYN pathology associated with altered phase separation. aSYN variants with decreased condensate formation lead to a redistribution of synaptic aSYN, which could result in its aggregation in the cytosol, similar to what has been recently described for G51D mutant mice wherein fibril induced aggregation was found to be increased, compared to WT mice^51^. Additionally, these variants may lead to formation of toxic aSYN oligomers which has been reported previously^47,53–57^. We further aim to highlight that G51D and A53E aSYN variants, which decrease the intrinsic propensity of aSYN to phase separate, show atypical disease courses with early age of onset^58–63^. Taken together, our data demonstrate an unexpected dichotomy associated with the dysregulation of aSYN phase separation, which may differentially affect disease pathogenesis.

## Methods

### Plasmids

Constructs for aSYN-YFP and VAMP2 were used from previous studies^12^. Briefly, wild-type human full-length SNCA and VAMP2, encoding aSYN and VAMP2, were cloned from cDNA obtained from human neuroblastoma cells (SH-SY5Y) and inserted into the pEYFP-N1 and pMD2.G vector (Addgene #96808, #12259) with a C-terminal YFP and Flag-tag, respectively. Gibson assembly was performed upon PCR amplification (Q5 Hot start HiFi 2xMM, M0494S, 2xHiFi DNA Assembly MM, E2621S, NEB, Ipswich, US). aSYN A30P, A30G, E46K, G51D, A53T, A53E and E83Q; and VAMP2 1-96 were generated using KLD substitution (M0554S, NEB, Ipswich, US). 5HT6-YFP-Inpp5e was a gift from Takanari Inoue (Addgene plasmid # 96808; http://n2t.net/addgene:96808; RRID:Addgene_96808)^64^. pMD2.G was a gift from Didier Trono (Addgene plasmid # 12259; http://n2t.net/addgene:12259; RRID:Addgene_12259). 4xmts-mScarlet-I was a gift from Dorus Gadella (Addgene plasmid # 98818; http://n2t.net/addgene:98818; RRID:Addgene_98818)^65^. pLAMP1-mCherry was a gift from Amy Palmer (Addgene plasmid # 45147; http://n2t.net/addgene:45147; RRID:Addgene_45147)^66^. The PrSS-Halo-KDEL plasmid was generated in the Nixon-Abell lab through three-part Gibson assembly of a HaloTag protein with a flanking Prolactin signal sequence and KDEL retention motif into a standard N1 backbone^67^. mRFP1-N1 was a gift from Robert Campbell & Michael Davidson & Roger Tsien (Addgene plasmid # 54635; http://n2t.net/addgene:54635; RRID:Addgene_54635)^67^. mCherry-KDEL was created from the PrSS-Halo-KDEL plasmid exchanging Halo for mCherry using Gibson Assembly (Q5 Hot start HiFi 2xMM, M0494S, 2xHiFi DNA Assembly MM, E2621S, NEB, Ipswich, US). All organelle plasmids were gifts from Jonathan Nixon-Abell, University of Cambridge. pmD2G-mTurquoise-VAMP2-FLAG and pmD2G-mScarlet-VAMP2-FLAG plasmids were created inserting mTurquoise2 or mScarlet into the pmD2G VAMP2 Flag plasmid using Gibson Assembly (Q5 Hot start HiFi 2xMM, M0494S, 2xHiFi DNA Assembly MM, E2621S, NEB, Ipswich, US). mTurquoise2 and mScarlet-I were amplified from plasmids ITPKA-mTurquoise2 (Addgene plasmid # 137811; http://n2t.net/addgene:137811; RRID:Addgene_137811) and pmScarlet_Giantin_C1, (Addgene plasmid # 85048; http://n2t.net/addgene:85048; RRID:Addgene_85048) both gifts from Dorus Gadella^65,68^. pmD2G-mTurquoise2-VAMP2_1-96-FLAG was derived from pmD2G-mTurquoise-VAMP2-FLAG plasmid through KLD deletion (M0554S, NEB, Ipswich, US). All plasmids were verified by sequencing.

### Cell culture and transfection

HeLa cells were obtained from the European Collection of Cell Cultures (ECACC 93021013) and grown in Dulbecco’s modified Eagle’s Medium (DMEM) high glucose (Gibco, 31966-021) supplemented with 10% fetal bovine serum (FBS, Sigma, F7524), 20 mM HEPES (Sigma, H0887) and 1% Penicillin/Streptomycin (Sigma, P0781). Cells were grown at 37 °C in a humidified incubator with 5% CO_2_. Cells were tested for mycoplasma contamination using MycoStripTM (IvivoGen, rep-mys-20, Toulouse, France). Cells were plated at 10,000 cells/well in 8-well ibidi dishes (ibidi, 80807) for confocal imaging or 48-well plates (Greiner, 677 180) for IncuCyte experiments. Cells were transfected the following day using Fugene HD Transfection reagent according to the manufacturer’s protocol (Promega, E2311). Briefly, per reaction 12.5 μL OptiMEM (Gibco, 31985-062) were set up in 1.5 mL sterile Eppendorf tubes. A total of 250 ng of DNA and 0.75 μL of Fugene reagent were added and incubated for 15 min at room temperature. The transfection mix was added to the cells for 1 min and then topped up with 300 μL complete media. Cells were imaged the next day.

### Microscopy, IncuCyte, and image analysis

Confocal imaging of condensate formation in HeLa cells was performed on an LSM780 microscope (Zeiss, Oberkochen, Germany) using a 63x oil immersion objective. YFP fluorescence was excited with the 514 nm laser. To evaluate aSYN condensate dispersion 1,6-hexanediol (Sigma, 240117) was prepared as 6% stock solution in complete DMEM media and added to the cells in a 1:1 ratio after the first image was taken. For fluorescence recovery after photobleaching (FRAP) experiments, images were taken with the 63x oil immersion objective, 20x zoom, 128 × 128 pixel resolution, at an imaging speed of 60 ms/image. Three pre-bleach images were acquired before the ROI was bleached with 100 iterations at 100% laser power using the 514 nm laser. Fluorescence recovery was recorded for 100 cycles. FRAP analysis was performed in FIJI using the FRAP profiler v2 plugin (Hardin lab, https://worms.zoology.wisc.edu/research/4d/4d.html). Organelle co-localisation studies in HeLa cells were performed on an LSM780 or LSM880 microscope (Zeiss, Oberkochen, Germany) using a 63x oil immersion objective and Super-resolution (SR) Airyscan processing. YFP was excited with the 488 nm laser, mTurquoise was excited with the 405 nm laser, mCherry and mScarlet were excited using the 561 nm laser. For experiments with Halo-KDEL, Janelia Fluor® 646 HaloTag® Ligand (Promega, GA1120) was added to the cells at the time of transfection at 1:10000 dilution. HaloTag treated cells were subsequently imaged with the 633 nm laser. Time course imaging was performed using the Super-resolution (SR) Airyscan feature of the LSM 880 microscope. Profile plots were generated in FIJI^69^ and plotted using GraphPad Prism 10.4.1. Zen 2.3 (black edition) and Zen 2.6 (blue edition) were used for data collection. For quantitative experiments wherein we evaluated % of cells with condensates, Hela cells were plated in 48-well plates and imaged with the IncuCyte S3 (Essen BioScience, Newark, UK). Phase bright field and green fluorescence images were taken using a 20x objective at a 4-hour interval at 200 ms exposure. Condensate formation (% of cells showing condensate formation) was evaluated at 16 hours after transfection. IncuCyte 2021 A was used for data analysis. At least three biological replicates with three technical replicates each were analyzed blinded to the investigator. Experiments comparing levels of condensate formation by aSYN-YFP and aSYN-mScarlet were imaged on LSM980 confocal microscope (Zeiss, Oberkochen, Germany) using a 40x oil immersion objective. Condensate formation (% of cells with condensates) was evaluated at 24 hours after transfection. To quantify aSYN levels in HeLa cells, images taken on LSM 980 confocal microscope (Zeiss, Oberkochen, Germany) using a 63x oil immersion objective were anonymized for blinded analysis using the File Name Encrypter module of the Blind Analysis Tools plugin (https://imagej.net/plugins/blind-analysis-tools) on FIJI. The mean fluorescence intensity of individual cells was measured and cytosolic aSYN levels were measured in three similarly sized regions within the cytosolic area.

### Immunocytochemistry

The following immunocytochemistry protocol was used for both HeLa cells and iPSC-derived neurons. Cells were fixed using a solution containing 4% paraformaldehyde and 4% sucrose in phosphate-buffered saline (PBS), pH 7.4 for 20 minutes at room temperature. Blocking and permeabilization were performed using 10% FBS, 1% BSA and 0.3% TritonX-100 in PBS for 1 h. Cells were stained with the corresponding primary antibodies (refer to Supplementary Table 1 for list of antibodies and dilutions used) in PBS containing 1% BSA, and incubated overnight at 4 °C. The following day, secondary antibodies at 1:1000 in PBS with 1% BSA was added to the cells and incubated at room temperature for 1 h. Subsequently, the cells were imaged on an LSM780 or LSM980 confocal microscope (Zeiss, Oberkochen, Germany). For pS129 staining experiments in HeLa cells, aSYN-YFP was excited using the 514 nm laser and pS129 labelled with secondary antibody Alexa Fluor 594 was excited with the 561 nm laser. The ratio of pS129 to total aSYN intensity in the condensates was measured using FIJI^69^. Briefly, a mask was created for the aSYN-YFP channel to select the condensates, which was then applied to the pS129 channel to obtain fluorescence intensities specifically in the condensates. For analysis of whole cell pS129 intensity, ROIs (regions of interest) were drawn to select individual cells and total pS129 and aSYN-YFP intensity was measured. iPSCs stained for pluripotency markers were treated with Alexa Fluor 488 secondary antibody and subsequently imaged with the 488 nm laser. Finally, endogenous alpha-synuclein and β-tubulin in iPSC neurons were stained with secondary antibodies Alexa Fluor 488 and Alexa Fluor 594 respectively and subsequently imaged with the 488 nm and 561 nm laser.

### Protein expression and purification of aSYN

Recombinant human full-length aSYN was expressed in BL21(DE3) competent Escherichia coli (C2527, NEB, Ipswich, US) using vector pET28a (Addgene #178032). aSYN A30P, A30G, E46K, G51D, A53T, A53E and E83Q were generated using KLD substitution (M0554S, NEB, Ipswich, US). Bacteria were cultured in LB media supplemented with 50 μg/mL kanamycin (37 °C, constant shaking at 250 rpm). Expression was induced at an OD600 of 0.8 using 1 mM isopropyl β-D-1-thiogalactopyranoside (IPTG) and cultured overnight at 25 °C. Cell pellets were harvested by centrifugation at 4000 × *g* for 30 min (AVANTI J-26, Beckman Coulter, USA). aSYN was purified using a protocol previously described^70^. Briefly, the cell pellet was resuspended in lysis buffer (10 mM Tris, 1 mM EDTA, Roche cOmplete EDTA free protease inhibitor cocktail, pH 8). The cells were disrupted using a cell disruptor (Constant Systems, Daventry, UK) and were ultra centrifuged at 4 °C, 186,000 × *g* for 20 min (Ti-45 rotor, Optima XPN 90, Beckman Coulter, USA). The supernatant was collected and heated for 20 min at 70 °C to precipitate heat-sensitive proteins, followed by ultracentrifugation as above. Streptomycin sulphate (5711, EMD Millipore, Darmstadt, Germany) was added at a final concentration of 10 mg/mL to the supernatant and continuously stirred at 4 °C for 15 min to precipitate DNA, followed by ultracentrifugation as above. Ammonium sulphate (434380010, Thermo Scientific) was added at a final concentration of 360 mg/mL to the supernatant and continuously stirred at 4 °C for 30 min to precipitate the protein. The precipitated protein was then centrifuged at 500 × *g* for 15 min, dissolved in 25 mM Tris, pH 7.7, and dialyzed overnight against the same buffer to remove salts. The protein was purified using ion exchange on a HiTrapTMQ HP 5 mL anion exchange column (17115401, Cytiva, Sweden) using gradient elution with 0-1 M NaCl in 25 mM Tris, pH 7.7. The collected protein fractions were run on SDS-PAGE and pooled fractions were further purified using size-exclusion chromatography on a HiLoadTM 16/600 SuperdexTM 75 pg column (28989333, Cytiva, Sweden). The fractions were collected, and their purity was confirmed using SDS-PAGE analysis. Protein concentrations were determined by measuring absorbance at 280 nm using an extinction coefficient of 5600 M^−1^cm^−1^. The monomeric protein was frozen in liquid nitrogen and stored in 25 mM HEPES buffer pH 7.4 at −70 °C. pET28a Cdk2ap1CAN was a gift from Lin He (Addgene plasmid # 178032; http://n2t.net/addgene:178032; RRID:Addgene_178032)^71^.

### Protein labelling

Labelling of proteins was performed in bicarbonate buffer (C3041, Sigma) at pH 8 using NHS-ester active fluorescent dyes. aSYN was labelled with AlexaFluor 488 5-SDP ester (A30052, Thermo Fisher Scientific). Excess-free dye was removed by buffer exchange using PD10 desalting columns (IP-0107-Z050.0-001, emp BIOTECH, Generon). Labelled protein concentrations were estimated using a molar extinction coefficient of ε494 nm = 72,000 M^−1^cm^−1^ for Alexa-488 5-SDP ester.

### Phase separation assays - turbidity measurements and confocal imaging

Phase separation assays were performed in 25 mM HEPES, pH 7.4 unless mentioned otherwise. Phase separation was induced by mixing aSYN and PEG 8000 (BP233-1, Fisher Bioreagent) in the presence of 2 mM calcium chloride (Sigma, 21108) as indicated respectively. **Turbidity measurements** - Phase-separated samples were set up as described above. The turbidity of the samples was measured at 350 nm, 25 °C using 96-well Greiner optical bottom plates on a CLARIOstar plate reader (BMG LABTECH, Ortenberg, Germany) under quiescent conditions. CLARIOStar 5.01 was used for data acquisition. A sample volume of 100 μL was used, and readings were taken within 5 minutes of sample preparation. For phase diagrams, raw turbidity data are plotted with background subtraction. Data were obtained from four independent replicates and were plotted using GraphPad Prism 10.4.1. **Confocal Microscopy** - Imaging of aSYN condensates in solution or in the presence of SUV or GUVs was performed on an LSM710, 780, 880 or 980 microscope (Zeiss, Oberkochen, Germany) using a 63x oil immersion objective. Alexa 488 was excited with the 488 nm laser, Cy5 was excited using the 633 nm laser, the T-PMT channel was used to image unlabelled aSYN. Images were taken using aSYN supplemented with 1% Alexa 488 labelled aSYN, or unlabelled aSYN for control experiments. Lipids were labelled incorporating 0.1% Cy5-PE. Zen 2.3 (black edition) and Zen 2.6 (blue edition) were used for data collection. Image analysis was performed in FIJI^69^. To analyze the number of condensates a mask for the droplets was generated, followed by particle analysis. Pearson correlation coefficients were calculated using the ColocFinder Plugin (https://imagej.nih.gov/ij/ plugins/colocalization-finder.html).

### Circular dichroism spectroscopy

Circular dichroism (CD) spectra were recorded on a Jasco J-810 Spectrapolarimeter (Jasco, Pfungstadt, Deutschland). aSYN was buffer exchanged into 20 mM sodium phosphate buffer pH 6.26 and incubated at 0.2 mg/mL protein concentration with an increasing amount of SUVs from 0, 0.1, 0.2, 0.3, 0.5 to 1 mM. SUVs were prepared as outlined below, containing 100% POPS, to enable high aSYN binding. Samples were mixed, briefly incubated and measured in a 1 mm pathlength quartz cuvette (QS High Precision Cell, 110-1-40, Hellma Analytics, Southend on Sea, United Kingdom). Spectra were recorded between 200 to 250 nm, with a 0.1 nm step, 1 nm bandwidth, and a speed of 50 nm/minute, averaged over 10 scans. Measurements were performed at room temperature.

### Preparation of small unilamellar vesicles and giant unilamellar vesicles

10 mM stock solutions of small unilamellar vesicles (SUVs) and giant unilamellar vesicles (GUVs) were prepared using a phospholipid mixture of DOPC (Avanti, 850375), DOPS (Avanti, 840035 C), cholesterol (Avanti 700000), and Cy5-PE (Avanti, 810335 C) in a 60:30:10:0.1 molar ratio. For SUV preparation, chloroform solutions of the lipids were combined in the required proportions and dried under a nitrogen stream forming a lipid film. The lipid film was hydrated with 25 mM HEPES buffer, pH 7.4 for 2 h with intermittent vortexing. For circular dichroism (CD) measurements, lipids were hydrated in 20 mM sodium phosphate buffer, pH 6.26. The resulting lipid suspensions were then subjected to 10 freeze-thaw cycles, using liquid nitrogen and a preheated water bath at 42 °C. SUVs were obtained by 2 min probe sonication, 10 sec on; 15 sec off, 20% amplitude (Sonic Dismembrator, Fisherbrand Model 505, Loughborough, UK). Turbidity measurements with SUVs were carried out with 80 µM aSYN and 1 mM lipids unless mentioned otherwise. Turbidity values were subtracted with the respective SUV control containing 1 mM SUV, 2 mM calcium and 15 % PEG 8000 to correct for calcium-induced aggregation of SUVs. GUVs were prepared using a gel-assisted swelling method as previously described^72^. Briefly, glass slides were coated with 100 µL of a 5% polyvinyl alcohol (PVA) solution (Sigma, 363065) and heated at 50 °C for 20 min to form a uniform PVA film. The required amount of lipids was then applied to the PVA-coated surface and dried under a gentle nitrogen stream. The PVA film was subsequently scraped and the lipid film was hydrated with 25 mM HEPES buffer, pH 7.4 for 2 h, resulting in the formation of GUVs.

### Yeast experiments

aSYN constructs were generated using site-directed mutagenesis carried out using the QuickChange II Site-Directed Mutagenesis Kit (Agilent Technologies, SC, USA) according to the manufacturer’s protocol in the plasmid backbone p426GPDpr encoding SNCA(WT)-GFP. The plasmid without aSyn, p426GPDpr-GFP was constructed by inserting the GFP coding sequence as a SpeI-XhoI digested PCR product. All constructs were confirmed by DNA sequencing. Yeast transformation was performed by standard lithium acetate method as described previously^73^. Supplementary Table 2 lists the plasmids and yeast strains used in this study. Cells were grown overnight in an orbital shaker, at 30 °C, 180 rpm, in yeast minimal synthetic defined (SD) medium (Takara Bio, Shiga, Japan), supplemented with drop-out mix (Takara Bio, Shiga, Japan) lacking the amino acid uracil (SD-URA) for transformant selection in a ratio of flask volume/medium of 5:1. Intracellular distribution of aSYN was evaluated by fluorescence microscopy, images were acquired using a Zeiss Axio Observer microscope (Zeiss, Oberkochen, Germany) equipped with a 100x oil objective.

### iPSC culture and differentiation into cortical neurons

The GM29370 iPSC line with a stable integration of Tet-On human NGN2 at the AAVS1 locus of the WTC-11 line was obtained from NIGMS Human Genetic Cell Repository at the Coriell Institute for Medical Research. iPSCs were cultured in StemFlex media (Gibco, A3349401) according to manufacturer’s guidelines on Matrigel (Corning, 354230) coated plates. 150 μL Matrigel was mixed with 15 mL DMEM/F12 media (Invitrogen, 10565018) and added to plates, which were then incubated at 37 °C for an hour before use. Cells were regularly passaged using Versene (Gibco, 15040066) for dissociation of iPSCs as clusters of cells or using Accutase (Gibco, A1110501) for single cell suspensions. Chroman 1 (Tocris, 7163) was used to inhibit ROCK signalling and maintain viability of iPSCs whenever cells were passaged with Accutase. At the start of differentiation (D0), iPSCs were passaged with Accutase and plated in Neural Induction media containing DMEM/F12 with Glutamax (Gibco, 10565018), 1X MEM-Non Essential Amino Acids (Gibco, 11140050) and 1X N-2 supplement (Gibco, 17502048). NGN2 expression was induced by adding Doxycycline (DOX) (Sigma, D9891) at 2 μg/ml. On D1 and D2, fresh Neural Induction media supplemented with DOX was added to the cells. On D3, cells were split with Accutase and resuspended in complete Neuronal medium containing Neurobasal Plus, supplemented with 1X B27 plus (Gibco, A3653401), BDNF 10 ng/mL (Gibco, AF-450-02-10UG), NT-3 10 ng/mL (Gibco, 450-03-10UG), Laminin 1 μg/mL (Sigma, L2020-1MG), DOX 2 μg/mL. The cell suspension was plated on 0.01% poly-L ornithine (Sigma, P4957) coated dishes (37 °C, overnight) at a density of 12,500 cells per well in an 8-well ibidi dish (ibidi, 80807) for live-cell imaging or 375,000 cells per well in a 12 well plate for western blot experiments. The neurons were subsequently allowed to mature in culture for 3-4 weeks with half-media changes every alternate day. DOX was withdrawn after D3. All experiments were performed between D21- D30.

### CRISPR-editing to generate SNCA-KO iPSC line

The online tool CRISPOR (https://crispor.gi.ucsc.edu/) was used to generate guides targeting Exon 2 of human SNCA^74^. Sense and antisense oligonucleotides encoding SNCA_G4_FOR (GTGCTGCTGAGAAAACCAAAC) and SNCA_G4_REV (GTTTGGTTTTCTCAGCAGCAC) were designed with 5’ CACC- and 5’ AAAC- overhangs respectively and were ordered from Merck. Oligos were annealed using a protocol with a slow gradient drop in temperature from 95 °C to 25 °C. The annealed oligos were ligated into pSpCas9(BB)−2A-GFP (PX458) vector (Addgene #48138) digested with BbsI restriction enzyme, followed by transformation into chemically competent NEB Stable E.coli (NEB, C3040H). pSpCas9(BB)−2A-GFP (PX458) was a gift from Feng Zhang (Addgene plasmid # 48138; http://n2t.net/addgene:48138; RRID:Addgene_48138)^75^. Colonies with the correct incorporation of the annealed guides into the vector (PX458-SNCA-G4-GFP) were identified through Sanger sequencing.

iPSCs were plated on 6 well plates at a density of 1 million cells per well in Stemflex media (Gibco, A3349401), supplemented with a CEPT cocktail composed of: Chroman 1 50 nM (Tocris, 7163), Emricasan 5 μM (Tocris, 7310/5), Polyamine supplement 1X (Sigma, P8483-5ML) and Trans-ISRIB 0.7 μM (Tocris, 5284/10)^76^. Four hours following plating, the cells were transfected with 2 μg of PX458-SNCA-G4-GFP plasmid using the Lipofectamine STEM transfection reagent according to the manufacturer’s protocol (Invitrogen, STEM00001). Three days following transfection, the GFP positive cells were sorted as single cells into 96 well plates through FACS using a BD Influx Cell Sorter (BD Biosciences, USA). The plates were incubated for ten days to allow for clonal expansion from single cells, with media changes every three days. The iPSC colonies were then collected for sequence analysis and subsequent expansion. PCR reactions were setup with forward primer TGGAAAAGCAAACAGTCGCA and reverse primer ACCCTTTAGACCCCGGGAAT to amplify a 700 kb fragment surrounding the expected edit site at Exon 2 of the SNCA gene and submitted for Sanger sequencing with an internal sequencing primer TCCGTGGTTAGGTGGCTAGA. iPSC clones with a homozygous deletion were selected for downstream analysis (SNCA-KO). WT and SNCA-KO iPSCs were characterized by immunocytochemistry for pluripotency markers (Supplementary Fig. 6a). Successful knockout of SNCA in the selected clone was validated by western blotting and immunocytochemistry of neurons 21 days after differentiation (Supplementary Fig. 6b, c).

### Preparation of lentiviral constructs

A third generation lentiviral FSW-jGCAMP8f vector (Addgene #197034) was used as a vector backbone to generate all lentiviral constructs used in this study. The FSW-jGCAMP8f plasmid encodes a human Synapsin promoter upstream and a WPRE sequence downstream of the gene of interest, respectively. jGCAMP8f was replaced with WT-aSYN mScarlet and a unique restriction site XbaI was added upstream of the WPRE sequence using Gibson Assembly to enable restriction enzyme based isolation of the vector backbone. The subsequent engineered plasmid was digested with BamHI and XbaI to isolate the vector backbone. Other aSYN variants tagged to mScarlet-I and Synaptophysin-mEmerald were amplified by PCR with overhanging sequences including the BamHI and XbaI restriction sites and subsequently cloned downstream of the human Synapsin promoter in the FSW vector using Gibson assembly (NEB, E2621S). Synaptophysin fragment was amplified from cDNA derived from SH-SY5Y cells. mScarlet-I was derived from pmScarlet_Giantin_C1 (Addgene # 85048), mEmerald was derived from mEmerald-Sec61b-C1 (Addgene # 90992). The assembled vectors were verified through sequencing and transfected into HEK293FT cells along with helper plasmids pMDLg/pRRE (Addgene #12251), pRSV-Rev (Addgene #12253), and pMD2.G (Addgene #12259) for viral packaging. Lipofectamine 3000 (Invitrogen, L3000008) was used for transfection according to the manufacturer’s protocol. The following day, the Lipofectamine-containing media was replaced with fresh media supplemented with ViralBoost Reagent (Alstem, VB100) at 2 μL/mL. Supernatants containing viral particles were collected 72 h after transfection and concentrated using Lenti-X™ Concentrator (Takara Bio, 631231). Viral titers were estimated with a Lentivirus qPCR Quantification Kit (Abcam, ab289841). iPSC-derived neurons were transduced with lentivirus at MOI 3 for aSYN-mScarlet variants and MOI 6 for Synaptophysin-mEmerald between D20-D24 after starting neuronal differentiation and incubated for five days before proceeding to confocal microscopy. For western blotting experiments, aSYN-mScarlet variants were transduced at MOI 1 and incubated for five days before lysis. FSW_jGCaMP8f was a gift from Ronald Hart (Addgene plasmid # 197034; http://n2t.net/addgene:197034; RRID:Addgene_197034). pmScarlet_Giantin_C1 was a gift from Dorus Gadella (Addgene plasmid # 85048; http://n2t.net/addgene:85048; RRID:Addgene_85048)^68^. mEmerald-Sec61b-C1 was a gift from Jennifer Lippincott-Schwartz (Addgene plasmid # 90992; http://n2t.net/addgene:90992; RRID:Addgene_90992)^77^. Plasmids pMDLg/pRRE (Addgene #12251; http://n2t.net/addgene:12251; RRID:Addgene_12251)^78^, pRSV-Rev (Addgene #12253; http://n2t.net/addgene:12253; RRID:Addgene_12253)^78^, and pMD2.G (Addgene #12259 encoding VSV-G; http://n2t.net/addgene:12259; RRID:Addgene_12259) were gifts from Didier Trono.

### Confocal microscopy of iPSC-derived neurons

iPSC-derived neurons transduced with aSYN-mScarlet and Synaptophysin-mEmerald were imaged live on an LSM980 confocal microscope (Zeiss, Oberkochen, Germany) using a 40x oil objective. mScarlet and mEmerald were excited with the 561 nm and 488 nm lasers, respectively. Images were obtained at 2x Nyquist sampling, 2x zoom and processed post-acquisition using the LSM Plus (deconvolution) processing feature to generate 16-bit images. For experiments using 1,6 hexanediol, a stock solution of 6% 1,6 hexanediol was prepared in complete media and added to the neurons to reach a final concentration of 3%. Images were obtained at 2 and 4 min post treatment, after which hexanediol containing media was replaced with fresh media to monitor recovery of synaptic enrichment at 2 and 4 min. FRAP assays on aSYN-mScarlet were performed over a time course of 250 cycles using a 40x objective at 6x zoom, 128 × 128 pixel resolution, with a frame time of 117.9 ms. The 561 nm laser was used for bleaching at 100% laser intensity over 25 iterations. Quantification of fluorescence recovery kinetics was performed using the FRAP profiler plugin on FIJI^69^.

### Quantification and statistical analysis

Data analysis and statistical analysis was performed using Excel 2016 and GraphPad Prism 10.4.1. All data are represented as mean ± standard error (s.d.) if not stated otherwise. Statistical analysis was carried out using unpaired two-tailed t-test, One-way ANOVA and Two-way ANOVA with Dunnett’s multiple comparison test, respectively as indicated. For 1,6-hexanediol experiments in HeLa cells RM Two-way ANOVA with Geisser-Greenhouse correction and Dunnett’s multiple comparisons test was performed. For 1,6-hexanediol experiments in neurons One-way ANOVA with Geisser-Greenhouse correction and Tukey’s multiple comparisons test was performed. Statistical parameters are reported in the Figures and the corresponding Figure Legends. Exact *p* values are shown throughout the manuscript. Data distribution was assumed to be normal, but this was not formally tested. No statistical methods were used to pre-determine sample sizes, but sample sizes are similar to those reported in previous publications^42,49,79^. Samples were randomly allocated into experimental groups. Data collection and analysis have been performed blinded when indicated. Data were included if the control (wild type) showed appropriate condensate formation.

### Reporting summary

Further information on research design is available in the Nature Portfolio Reporting Summary linked to this article.

## Supplementary information

Supplementary Information

Description of Additional Supplementary Files

Supplementary Video 1

Supplementary Video 2

Reporting Summary

Transparent Peer Review file

## Source data

Source Data

## Data Availability

Source data are provided with this study. Plasmids generated in this study are available with a completed material transfer agreement. All data supporting the findings of this study are available on request. Source data are provided with this paper.
