# Supplementary material for: Dual effect of α-synuclein disease variants on condensate formation
Source: Nat Commun. 2026 Sep 2;17:10442. doi: 10.1038/s41467-026-77002-4 (PMC13631337; doi:10.1038/s41467-026-77002-4)
Supplement: Supplementary file 1 [file 41467_2026_77002_MOESM1_ESM.pdf]

## Supplementary Information:

### Supplementary Figure 1

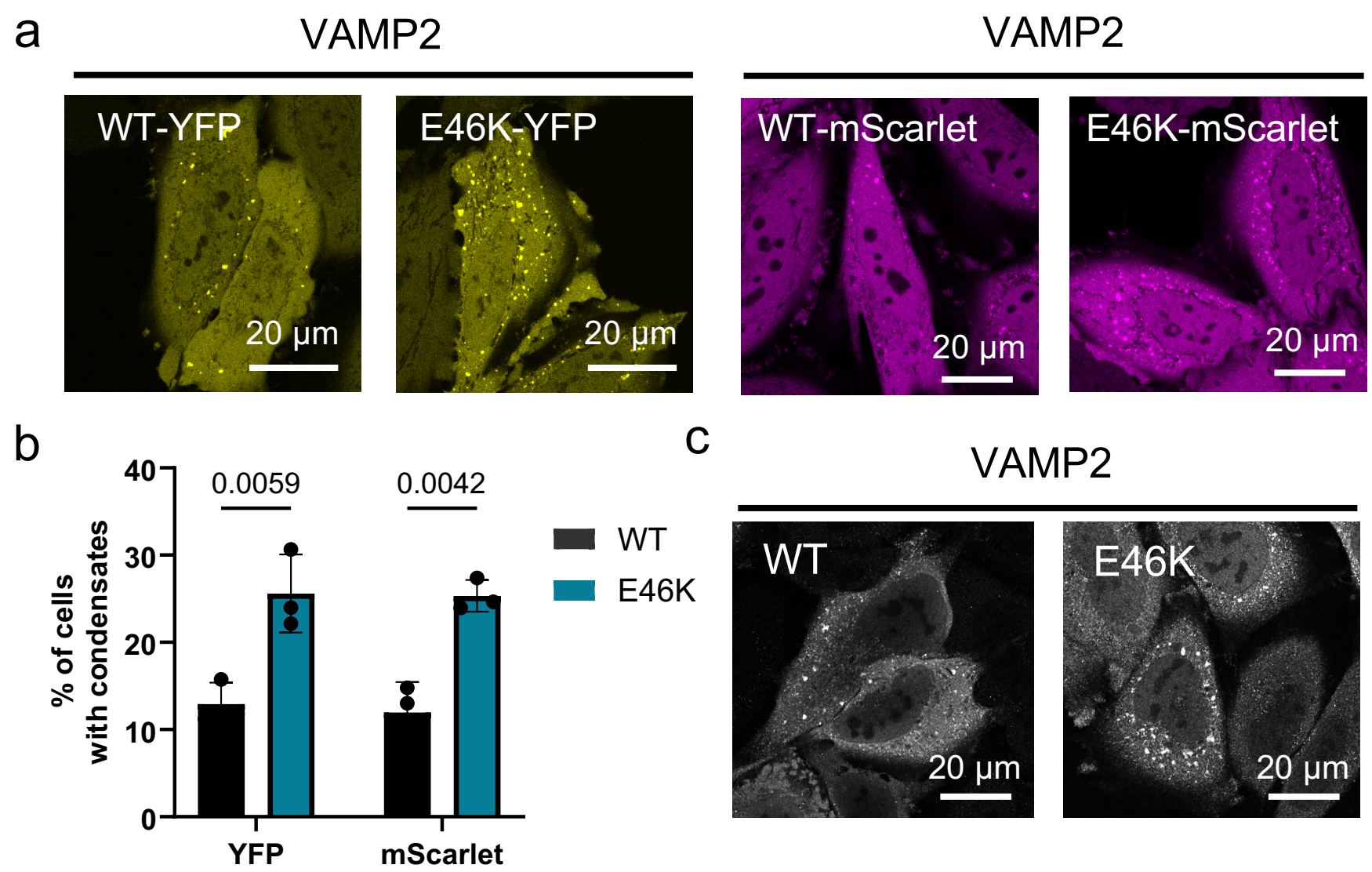


**Supplementary Figure 1. Effect of fluorescent protein tags on aSYN condensate formation in cells**

(a) Confocal imaging showing condensate formation in HeLa cells co-expressing YFP- or mScarlet-tagged aSYN WT and E46K with VAMP2.

(b) Quantification of condensate formation in cells by aSYN WT or E46K- tagged with YFP or mScarlet, n indicates 3 biological replicates. Data are shown as mean ± s.d. Two-way ANOVA with Tukey's multiple comparisons test. Significant p values are: p=0.0059 for WT-YFP vs E46K-YFP, p=0.0067 for WT-YFP vs E46K-mScarlet, p=0.0037 for E46K-YFP vs WT-mScarlet, p=0.0042 for WT-mScarlet vs E46K-mScarlet.

(c) Confocal imaging showing condensate formation in HeLa cells co-expressing untagged aSYN WT and E46K with VAMP2. Imaged after immunocytochemistry with anti alpha-synuclein (SYSY 128208) antibody.

## Supplementary Figure 2

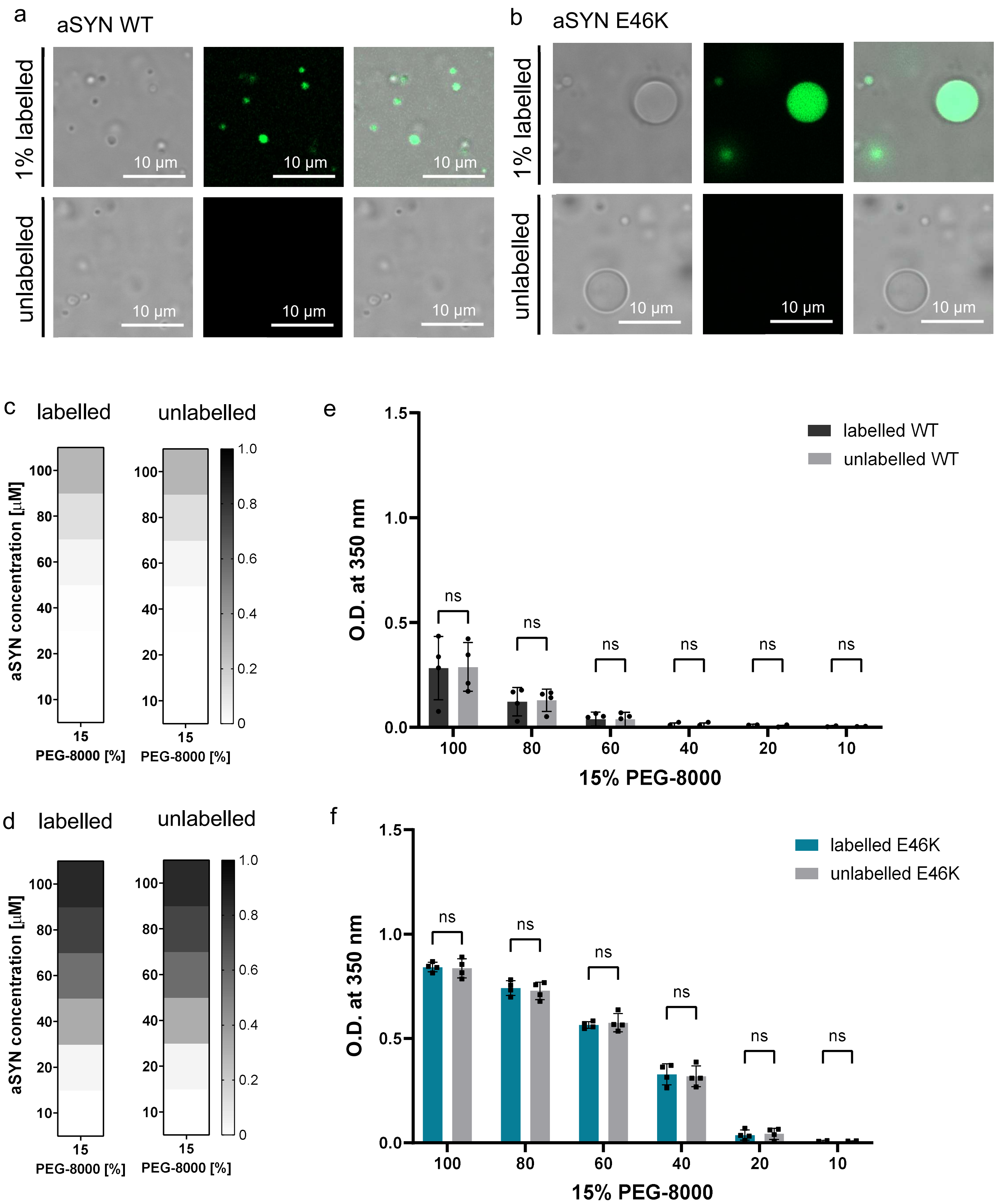

**Supplementary Figure 2. aSYN condensate formation in vitro - comparison with unlabelled protein**

(a/b) Confocal imaging showing aSYN phase separation in the presence of 2 mM $Ca^{2+}$ and 15% PEG 8000, 25 mM HEPES buffer pH 7.4. αSYN was used at 100 µM and was either supplemented with 1% aSYN-Alexa 488 or used as 100% unlabelled protein. In both conditions, aSYN shows comparable condensate formation.

(c/d) Quantification of aSYN phase separation *in vitro* using turbidity measurements (O.D. at 350 nm) shown as heatmaps for WT and E46K comparing aSYN with 1% aSYN-Alexa 488 and 100% unlabelled protein. All measurements were done in the presence of 2 mM $Ca^{2+}$ and 15% PEG 8000, 25 mM HEPES buffer pH 7.4. Data represent 4 independent replicates from two batches of recombinant aSYN.

(e/f) Quantification of turbidity data (O.D. at 350 nm) in (c/d). n represents 4 independent replicates from two batches of recombinant aSYN. Data are shown as mean ± s.d. Two-way ANOVA with Šídák's multiple comparisons test.

**Supplementary Figure 3:**

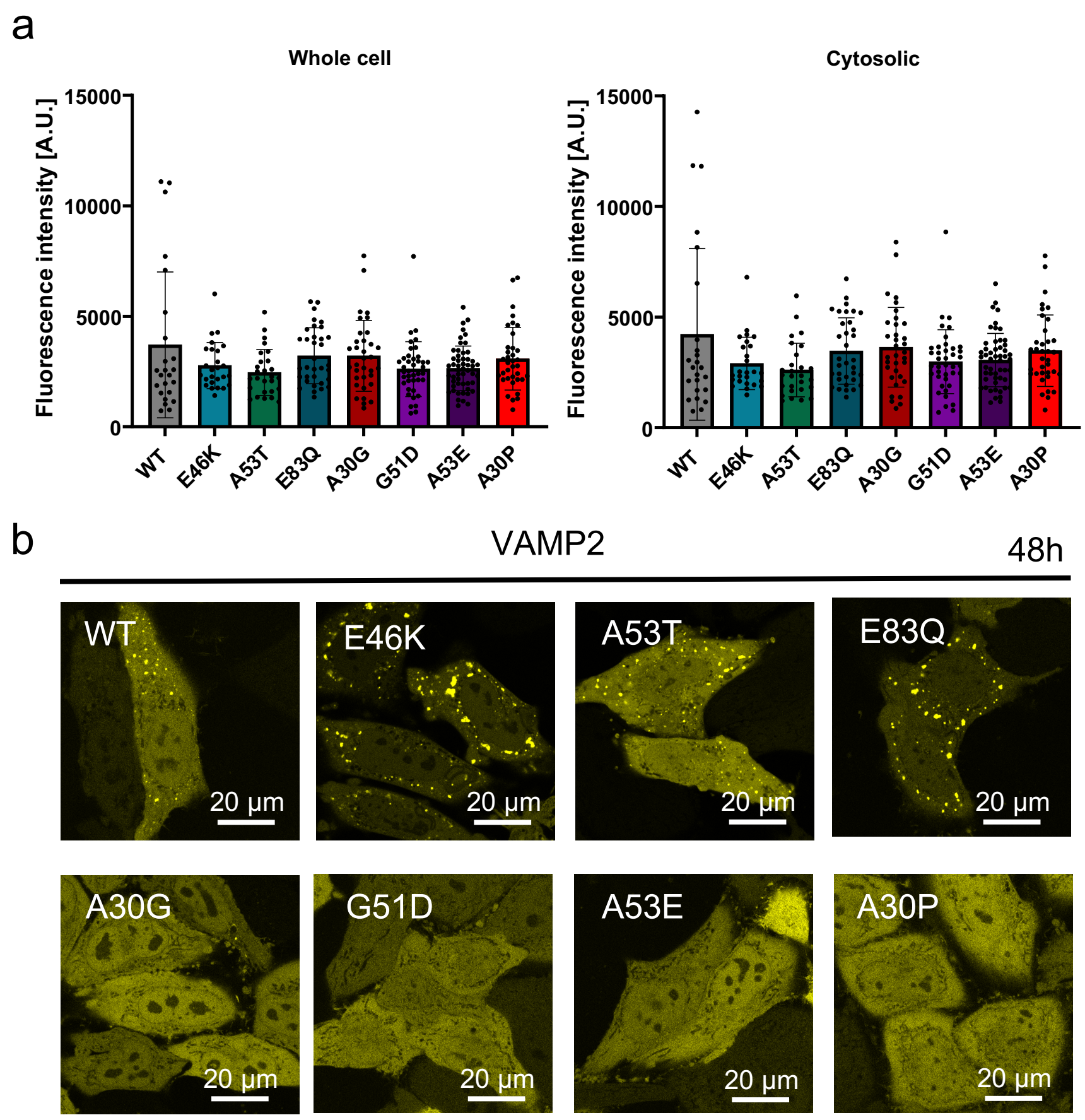


**Supplementary Figure 3. Expression levels of aSYN variants at 24 hours, and evaluation of condensate formation at 48 hours**

(a) aSYN-YFP intensity of whole cell (including condensate) and cytosolic aSYN-YFP across different aSYN variants upon VAMP2 co-expression imaged at 24 hours post transfection. n represents individual cells with from 3 biological replicates, n=24, 26, 27, 34, 34, 40, 52, 36 cells for WT, E46K, A53T, E83Q, A30G, G51D, A53E and A30P, respectively. Data are shown as mean ± s.d.

(b) HeLa cells co-expressing aSYN-YFP and VAMP2 for 48 hours, showing condensate formation for aSYN WT, E46K, A53T, and E83Q; but no condensate formation for A30G, G51D, A53E, and A30P.

**Supplementary Figure 4:**

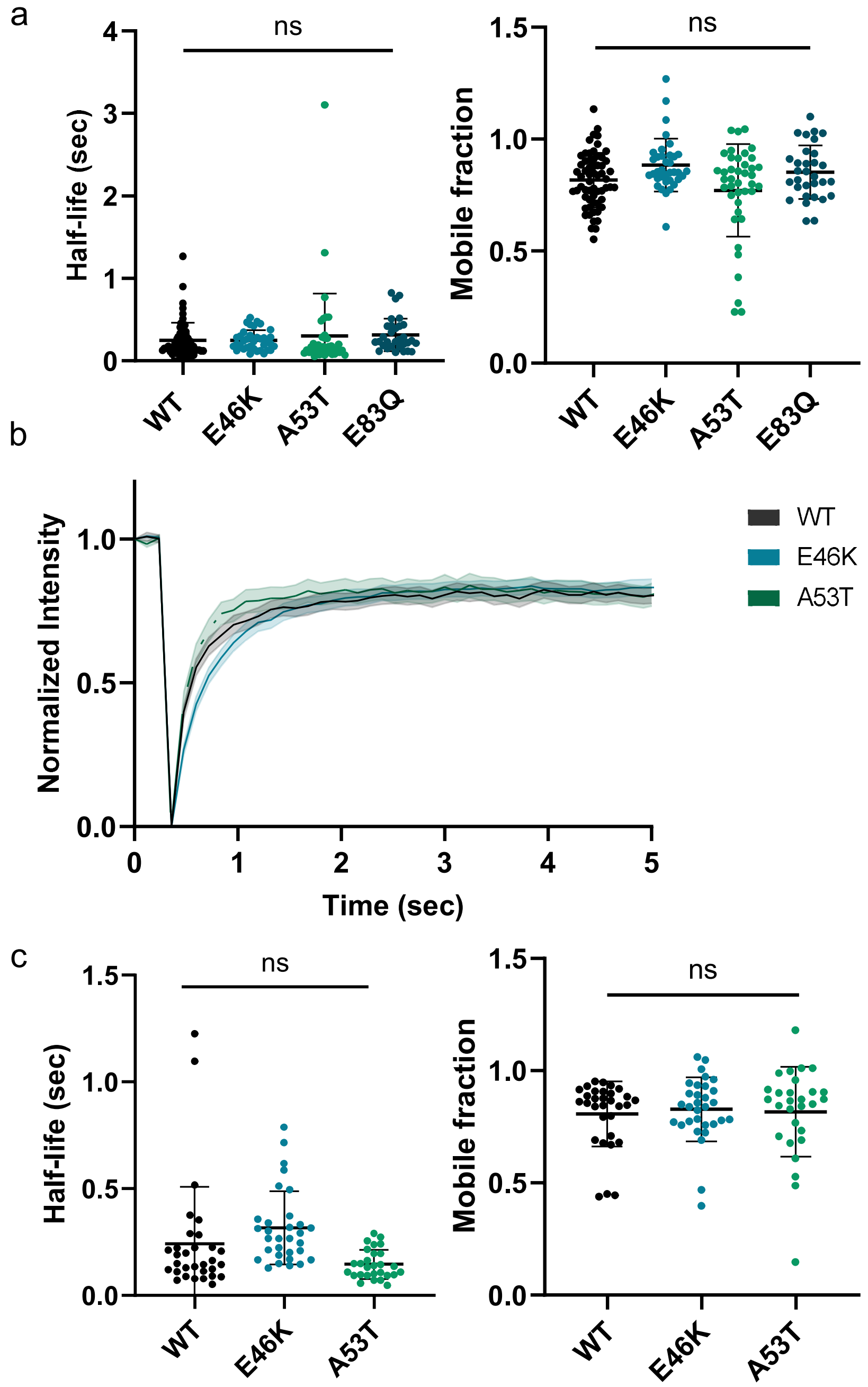

**Supplementary Figure 4. FRAP kinetics after 24 hours and 48 hours**

(a) Quantification of half-life and mobile fraction after curve fitting with a one phase decay equation for FRAP experiments shown in Figure 3(a), at 24 hours post transfection. n represents number of condensates measured from a minimum of 4 biological replicates, with n=61, 36, 40, 31 for WT, E46K, A53T, E83Q, respectively. Data are shown as mean ± s.d. One-way ANOVA with Dunnett's multiple comparison test.

(b) FRAP experiments of aSYN condensates at 48 hours post transfection showing aSYN recovery. n represents number of FRAP measurements from 3 biological replicates with n=31 for WT, n=31 for E46K, n=28 for A53T. Data are shown as mean ± s.e.m.

(c) Quantification of half-life and mobile fraction after curve fitting with a one phase decay equation for FRAP experiments shown in (b) at 48 hours post transfection. n represents number of FRAP measurements from 3 biological replicates with n=31 for WT, n=31 for E46K, n=28 for A53T. E46K shows a trend towards higher half-life, indicating slower recovery, however mobile fraction shows no change across variants. Data are shown as mean ± s.d. One-way ANOVA with Dunnett's multiple comparison test.

**Supplementary Figure 5:**

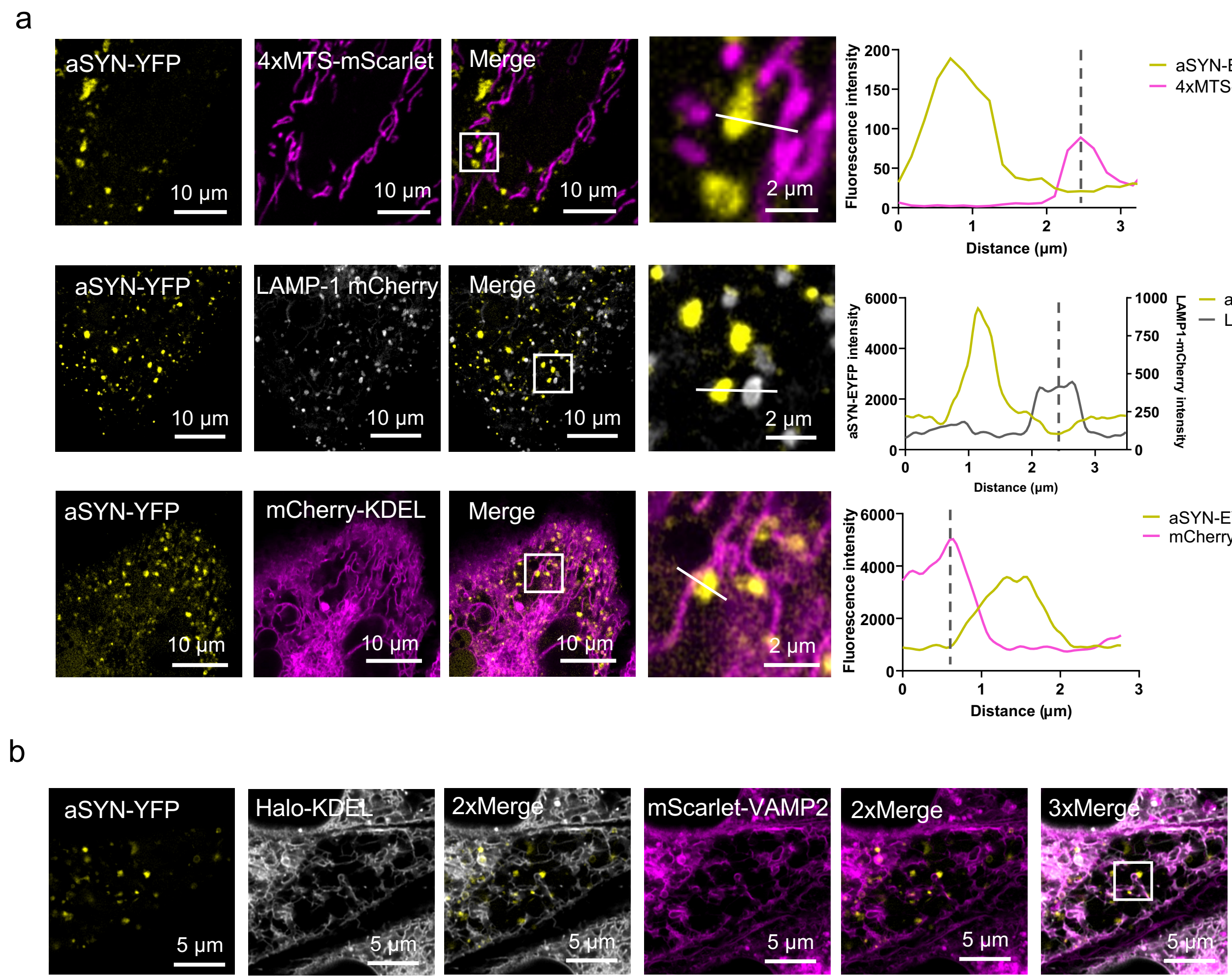


**Supplementary Figure 5. Colocalization of aSYN condensates with intracellular organelles, related to Main Figure 5**

(a) HeLa cells co-expressing aSYN-YFP, VAMP2 FL, and organelle markers 4X-MTS-mScarlet, LAMP1-mCherry or mCherry-KDEL for visualization of aSYN condensates on mitochondria, lysosomes or ER, respectively. Representative line diagrams of aSYN condensates and the corresponding organelles, indicating close association of aSYN condensates with the ER.

(b) Three-colour experiment in HeLa cells co-expressing aSYN-YFP, Halo-KDEL and mScarlet-VAMP2 FL showing aSYN condensates on VAMP2 positive ER tubules. Also shown are merged

images of aSYN-YFP with Halo-KDEL, aSYN-YFP with mScarlet-VAMP2 and a three-colour merge with aSYN-YFP, Halo-KDEL and mScarlet-VAMP2.

**Supplementary Figure 6:**

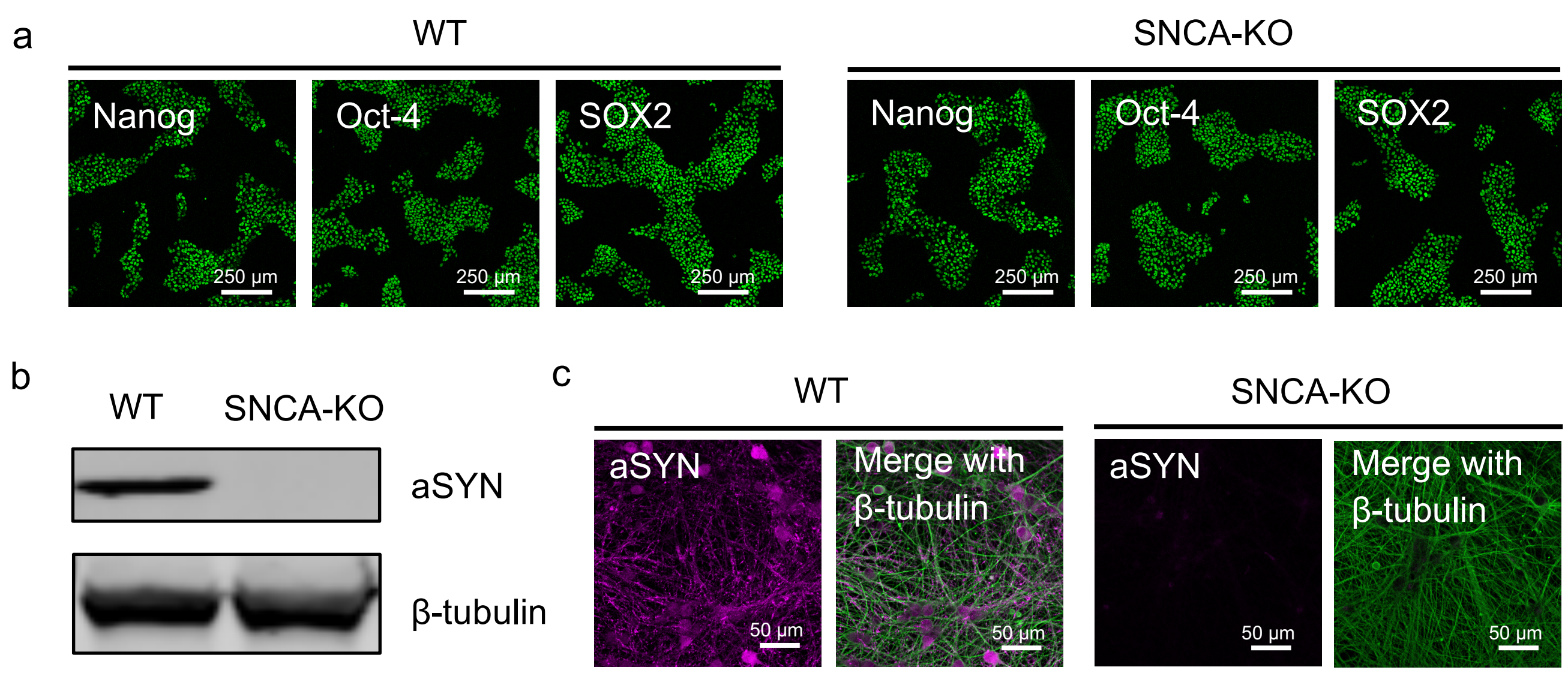


**Supplementary Figure 6.**

**Characterization of iPSC-derived neurons, related to Main Figure 6**

(a) Immunocytochemistry showing expression of stem cell pluripotency markers in WT and SNCA-knock out iPSCs.

(b) Western blot showing aSYN expression at D21 for neurons differentiated from WT and SNCA-knock out iPSCs.

(c) Immunocytochemistry for aSYN and β-tubulin at D21 for neurons differentiated from WT and SNCA-knock out iPSCs.

**Supplementary Figure 7:**

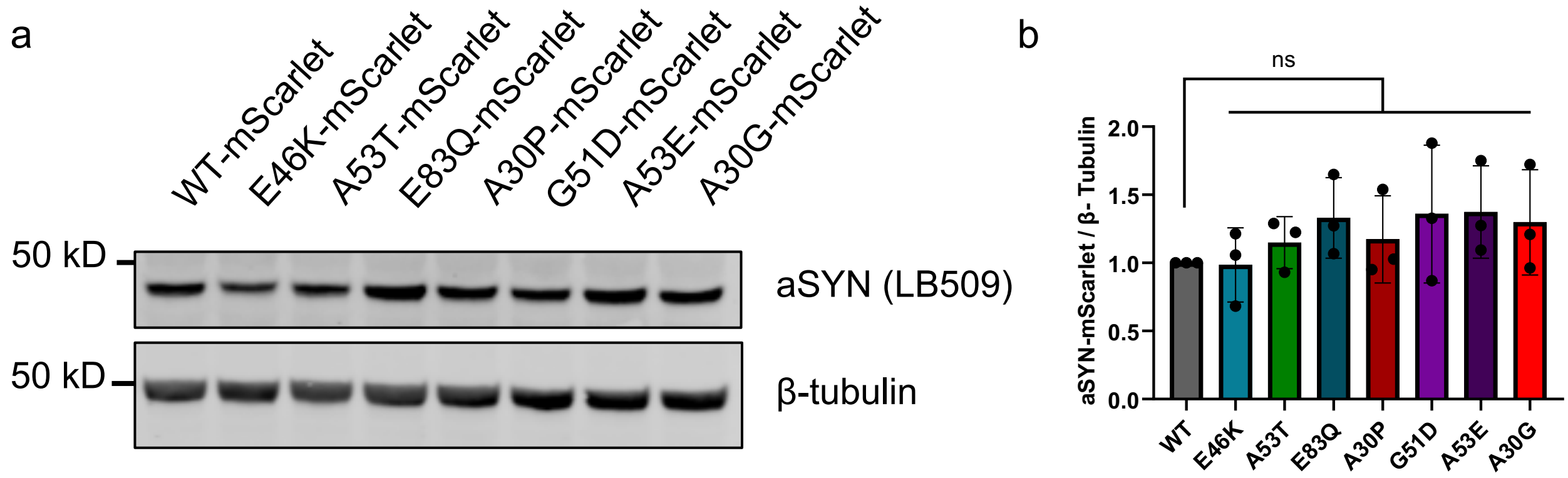


**Supplementary Figure 7. Expression levels of different aSYN-mScarlet variants in iPSC-derived neurons, related to Main Figure 6**

(a) aSYN-mScarlet expression levels in iPSC-derived neurons transduced with lentiviruses encoding different aSYN-mScarlet variants between D21-25 and lysed 5 days post transduction.

(b) Quantification of aSYN levels relative to β-tubulin. n indicates 3 biological replicates. Data are shown as mean ± s.d. One-way ANOVA with Dunnett's multiple comparison test. No significant differences are seen between any of the variants.

**Supplementary Videos**

**Supplementary Video 1**: Time lapse movie of WT aSYN-EYFP condensates moving along the ER network in HeLa cells. ER labelled with mCherry-KDEL. Related to Figure 5.

**Supplementary Video 2**: Time lapse movie of E46K aSYN-EYFP condensates moving along the ER network in HeLa cells. ER labelled with mCherry-KDEL. Related to Figure 5.

**Supplementary Table 1: List of antibodies used in this study**

| Antibody | Catalog No. | Application | Dilution |
|---|---|---|---|
| **Primary** | | | |
| Mouse anti alpha-Synuclein Phospho (Ser129), clone 81A | BioLegend, 825701 | ICC | 1:1000 |
| StemLight Pluripotency Transcription Factor Antibody Kit (Nanog, Oct4A, SOX2) | CST, 9093 | ICC | 1:1000 |
| Mouse anti alpha-Synuclein antibody | Synaptic Systems, 128211 | ICC | 1:250 |
| β3-Tubulin (D65A4) XP® Rabbit mAb | CST, 5666 | ICC | 1:500 |
| Rabbit anti Alpha-synuclein antibody | Synaptic Systems, 128208 | ICC | 1:250 |
| Mouse anti Alpha-synuclein Monoclonal Antibody (LB509) | Invitrogen, 180215 | WB | 1:1000 |
| β3-Tubulin (D65A4) XP® Rabbit mAb | CST, 5666 | WB | 1:1000 |
| **Secondary** | | | |
| F(ab')2-Goat anti-Mouse IgG (H+L), Alexa Fluor™ 488 | Invitrogen, A11017 | ICC | 1:1000 |
| F(ab')2-Goat anti-Mouse IgG (H+L), Alexa Fluor™ 594 | Invitrogen, A11020 | ICC | 1:1000 |
| F(ab')2-Goat anti-Rabbit IgG (H+L), Alexa Fluor™ 488 | Invitrogen, A11070 | ICC | 1:1000 |
| F(ab')2-Goat anti-Rabbit IgG (H+L), Alexa Fluor™ 594 | Invitrogen, A11072 | ICC | 1:1000 |
| Goat anti-Mouse IgG (H+L), DyLight™ 800 | Invitrogen, SA535521 | WB | 1:10000 |
| Goat anti-Rabbit IgG (H+L), DyLight™ 680 | Invitrogen, 35568 | WB | 1:10000 |

**Supplementary Table 2: List of yeast strains used in this study**

| Strain | Genotype | Source |
|---|---|---|
| BY4741 | *MATa his3Δ1 leu2Δ0 met15Δ0 ura3Δ0* | Euroscarf |
| BY4741 pGPD-GFP | BY4741 harbouring *p426GPDpr-GFP* | This Study |
| BY4741 pGPD-SNCA (WT) - GFP | BY4741 harbouring *p426GPDpr*-SNCA (WT)-GFP | 10.1126/science.1090439. |
| BY4741 pGPD-SNCA (A53T)-GFP | BY4741 harbouring *p426GPDpr*-SNCA (A53T)-GFP | 10.1126/science.1090439. |
| BY4741 pGPD-SNCA (A53E)-GFP | BY4741 harbouring *p426GPDpr*-SNCA (A53E)-GFP | This Study |
| BY4741 pGPD-SNCA (E46K)-GFP | BY4741 harbouring *p426GPDpr*-SNCA (E46K)-GFP | This Study |
| BY4741 pGPD-SNCA (A30G)-GFP | BY4741 harbouring *p426GPDpr*-SNCA (A30G)-GFP | This Study |
| BY4741 pGPD-SNCA (A30P)-GFP | BY4741 harbouring *p426GPDpr*-SNCA (A30P)-GFP | This Study |
| BY4741 pGPD-SNCA (G51D)-GFP | BY4741 harbouring *p426GPDpr*-SNCA G51D)-GFP | This Study |
