# Supplementary material for: Dual effect of α-synuclein disease variants on condensate formation
Source: Nat Commun. 2026 Sep 2;17:10442. doi: 10.1038/s41467-026-77002-4 (PMC13631337; doi:10.1038/s41467-026-77002-4)
Supplement: Supplementary file 2 [file 41467_2026_77002_MOESM2_ESM.pdf]

Description of Additional Supplementary Files

Supplementary Video 1: Time lapse movie of WT aSYN-EYFP condensates moving along the ER network in HeLa cells. ER labelled with mCherry-KDEL. Related to Figure 5.

Supplementary Video 2: Time lapse movie of E46K aSYN-EYFP condensates moving along the ER network in HeLa cells. ER labelled with mCherry-KDEL. Related to Figure 5.
