# Supplementary material for: Dual effect of α-synuclein disease variants on condensate formation
Source: Nat Commun. 2026 Sep 2;17:10442. doi: 10.1038/s41467-026-77002-4 (PMC13631337; doi:10.1038/s41467-026-77002-4)
Supplement: Supplementary file 3 [file 41467_2026_77002_MOESM5_ESM.pdf]

Corresponding author(s): Janin Lautenschlaeger

Last updated by author(s): Jul 13, 2026

Reporting Summary

Nature Portfolio wishes to improve the reproducibility of the work that we publish. This form provides structure for consistency and transparency in reporting. For further information on Nature Portfolio policies, see our Editorial Policies and the Editorial Policy Checklist.

Statistics

For all statistical analyses, confirm that the following items are present in the figure legend, table legend, main text, or Methods section.

n/a	Confirmed	
☐	☒	The exact sample size (*n*) for each experimental group/condition, given as a discrete number and unit of measurement
☐	☒	A statement on whether measurements were taken from distinct samples or whether the same sample was measured repeatedly
☐	☒	The statistical test(s) used AND whether they are one- or two-sided<br>*Only common tests should be described solely by name; describe more complex techniques in the Methods section.*
☒	☐	A description of all covariates tested
☐	☒	A description of any assumptions or corrections, such as tests of normality and adjustment for multiple comparisons
☐	☒	A full description of the statistical parameters including central tendency (e.g. means) or other basic estimates (e.g. regression coefficient) AND variation (e.g. standard deviation) or associated estimates of uncertainty (e.g. confidence intervals)
☐	☒	For null hypothesis testing, the test statistic (e.g. *F*, *t*, *r*) with confidence intervals, effect sizes, degrees of freedom and *P* value noted<br>*Give P values as exact values whenever suitable.*
☒	☐	For Bayesian analysis, information on the choice of priors and Markov chain Monte Carlo settings
☒	☐	For hierarchical and complex designs, identification of the appropriate level for tests and full reporting of outcomes
☐	☒	Estimates of effect sizes (e.g. Cohen's *d*, Pearson's *r*), indicating how they were calculated

Our web collection on statistics for biologists contains articles on many of the points above.

Software and code

Policy information about availability of computer code

Data collection	Data collection was performed using Zen 2.3 (black edition) and Zen 2.6 (blue edition), Incucyte 2021A, Incucyte 2022B Rev2, Clariostar 5.01, Image Studio 5.2.
Data analysis	Image analysis was performed uing FIJI, data analysis was performed using Excel 2016 and Prism 9.3.1

For manuscripts utilizing custom algorithms or software that are central to the research but not yet described in published literature, software must be made available to editors and reviewers. We strongly encourage code deposition in a community repository (e.g. GitHub). See the Nature Portfolio guidelines for submitting code & software for further information.

Data

Policy information about availability of data

All manuscripts must include a data availability statement. This statement should provide the following information, where applicable:

- Accession codes, unique identifiers, or web links for publicly available datasets
- A description of any restrictions on data availability
- For clinical datasets or third party data, please ensure that the statement adheres to our policy

Source data are provided with the study.

Research involving human participants, their data, or biological material

Policy information about studies with human participants or human data. See also policy information about sex, gender (identity/presentation), and sexual orientation and race, ethnicity and racism.

Reporting on sex and gender	N/A
Reporting on race, ethnicity, or other socially relevant groupings	N/A
Population characteristics	N/A
Recruitment	N/A
Ethics oversight	N/A

Note that full information on the approval of the study protocol must also be provided in the manuscript.

Field-specific reporting

Please select the one below that is the best fit for your research. If you are not sure, read the appropriate sections before making your selection.

[x] Life sciences [ ] Behavioural & social sciences [ ] Ecological, evolutionary & environmental sciences

For a reference copy of the document with all sections, see nature.com/documents/nr-reporting-summary-flat.pdf

Life sciences study design

All studies must disclose on these points even when the disclosure is negative.

Sample size	No statistical methods were used to pre-determine sample sizes but our sample sizes are similar to those reported in previous publications (Wu et al Mol Cell 2019, Wu et al Mol Cell 2021, Park et al Nat Comm 2021, Park et al Nat Comm 2023).
Data exclusions	Data were included if the control (wildtype) showed appropriate condensate formation.
Replication	All experiments have been replicated 2 or more times as indicated in the manuscript. All attempts of replication were successful.
Randomization	Samples were randomly allocated into experimental groups.
Blinding	Blinded analysis was performed for quantification of condensate formation in HeLa cells (Incucyte analysis) and for quantification of the levels of synaptic enrichment in iPSC-derived neurons. Image acquisition and analysis were performed non-blinded otherwise, but performed to an objectively defined standard ensuring reproducibility.

Reporting for specific materials, systems and methods

We require information from authors about some types of materials, experimental systems and methods used in many studies. Here, indicate whether each material, system or method listed is relevant to your study. If you are not sure if a list item applies to your research, read the appropriate section before selecting a response.

Materials & experimental systems

n/a	Involved in the study	
[ ]	[x]	Antibodies
[ ]	[x]	Eukaryotic cell lines
[x]	[ ]	Palaeontology and archaeology
[x]	[ ]	Animals and other organisms
[x]	[ ]	Clinical data
[x]	[ ]	Dual use research of concern
[x]	[ ]	Plants

Methods

n/a	Involved in the study	
[x]	[ ]	ChIP-seq
[x]	[ ]	Flow cytometry
[x]	[ ]	MRI-based neuroimaging

Antibodies

Antibodies used	1. Phosphoserine 129 (pS129) Alpha-Synuclein, clone 81A (Mouse Monoclonal) BioLegend Cat# 825701; AB_ 2564891, Lot B318449<br>2. StemLight Pluripotency Transcription Factor Antibody Kit (Nanog, Oct4A, SOX2) (Rabbit Monoclonal) Cell Signaling Technology Cat# 9093; AB_10697029, Lot 8-Nanog, Lot 8-Sox2, Lot 20-Oct-4<br>3. Alpha-Synuclein (Mouse Monoclonal) Synaptic Systems Cat# 128 211; AB_2619811, Lot 1-9

4. β3-Tubulin (Rabbit Monoclonal) Cell Signaling Technology Cat# 5666; AB_10691594, Lot 4
5. Alpha-synuclein (Rabbit Monoclonal) Synaptic Systems Cat 128 208; AB_3083018
6. Alpha-Synuclein, LB509 (Mouse Monoclonal) Invitrogen Cat# 180215; AB_2920932, Lot 2757052
7. F(ab')2-Goat anti-Mouse IgG (H+L) Cross-Adsorbed Secondary Antibody, Alexa Fluor™ 594 Invitrogen Cat# A11020; AB_2534087, Lot 2465084
8. F(ab')2-Goat anti-Rabbit IgG (H+L) Cross-Adsorbed Secondary Antibody, Alexa Fluor™ 488 Invitrogen Cat# A11070; AB_2534114, Lot 2434560
9. F(ab')2-Goat anti-Mouse IgG (H+L) Cross-Adsorbed Secondary Antibody, Alexa Fluor™ 488 Invitrogen Cat# A11017; AB_2534084, Lot 2335735
10. F(ab')2-Goat anti-Rabbit IgG (H+L) Cross-Adsorbed Secondary Antibody, Alexa Fluor™ 594 Invitrogen Cat# A11072; AB_2534116, Lot 2502896
11. Goat anti-Mouse IgG (H+L) Secondary Antibody, DyLight™ 800 Invitrogen Cat# SA535521; AB_2556774, Lot WA318906
12. Goat anti-Rabbit IgG (H+L) Secondary Antibody, DyLight™ 680 Invitrogen Cat# 35568; AB_614946, Lot WF328091

Validation

1. Phosphoserine 129 (pS129) Alpha-Synuclein, clone 81A (Mouse Monoclonal) BioLegend Cat# 825701- Manufacturer validation- Reacts with human pS129. Use for ICC reported in the literature (Volpicelli-Daley et al., Nature Protocols, 2014), but not verified in house. Antibody has been verified for IHC by the manufacturer using formalin-fixed paraffin-embedded immunohistochemistry of disease human brain tissue.

2. StemLight Pluripotency Transcription Factor Antibody Kit (Nanog, Oct4A, SOX2) (Rabbit Monoclonal) Cell Signaling Technology Cat# 9093- Manufacturer validation- All antibodies verified for western blotting, ICC and flow cytometry. Nanog and Sox2 react with human antigens only, Oct-4 recognizes human and mouse variants. Nanog and Sox2 antibodies were validated for ICC application as a marker for pluripotent cells using an NTERA-2 cell line, a pluripotent human embryonic carcinoma cell line as a positive control and HeLa cells as negative control. Oct-4 antibody was validated for ICC in NTERA2 and mouse embryonic stem (mES) cells.

3. Alpha-Synuclein (Mouse Monoclonal) Synaptic Systems Cat# 128 211- Manufacturer validation- Reacts with human, rat and mouse. Validated in ICC, IHC and WB applications. Use for ICC verified in rat hippocampal neurons. Specific to alpha-synuclein, no reactivity with beta and gamma synuclein.

4. β3-Tubulin (Rabbit Monoclonal) Cell Signaling Technology Cat# 5666- Manufacturer validation- Recognizes human, mouse and rat. Validated for WB, IP and IHC. Use for ICC not reported by manufacturer but has been reported in published papers (Granatiero et al., Autophagy, 2021). WB confirms neuron-specific expression in human cerebellum and rat brain, but not in HeLa and C6 cell lines.

5. Alpha-synuclein Monoclonal Antibody (LB509) Cat# 180215- Manufacturer validation- Recognizes human alpha-synuclein. Validated for immunohistochemistry by manufacturer. Use in WB and ICC reported in published studies (Oaks et al., PLOS One 2013, Feng-Li et al., Disease Models and Mechanisms, 2022).

Eukaryotic cell lines

Policy information about cell lines and Sex and Gender in Research

Cell line source(s)

HeLa cells were obtained from the European Collection of Cell Cultures (ECACC 93021013), sex: female, human cell line. The GM29370 (i3N) iPSC line with a stable integration of Tet-On human NGN2 at the AAVS1 locus of the WTC-11 line was obtained from NIGMS Human Genetic Cell Repository at the Coriell Institute for Medical Research, sex: male, human cell line. The SNCA-KO iPSC line was derived from the GM29370 line in house.

Authentication

All cells were validated morphologically. The iPSC lines (parental wildtype and the SNCA KO line) were tested for expression of pluripotency stem cell markers Sox2, Oct4 and Nanog.

Mycoplasma contamination

All cell lines were tested negative for mycosplasma using the Invivogen Mycostrip-Mycoplasma Detection Kit.

Commonly misidentified lines
(See ICLAC register)

No commonly misidentified lines per the ICLAC register were used.

Plants

Seed stocks

N/A

Novel plant genotypes

N/A

Authentication

N/A
