# Supplementary material for: Dual effect of α-synuclein disease variants on condensate formation
Source: Nat Commun. 2026 Sep 2;17:10442. doi: 10.1038/s41467-026-77002-4 (PMC13631337; doi:10.1038/s41467-026-77002-4)
Supplement: Supplementary file 4 [file 41467_2026_77002_MOESM6_ESM.pdf]

nature portfolio

Peer Review File

Dual effect of α-synuclein disease variants on condensate formation

Corresponding Author: Dr Janin Lautenschlaeger

This file contains all reviewer reports in order by version, followed by all author rebuttals in order by version.

Version 0:

Reviewer comments:

Reviewer #1

(Remarks to the Author)
The authors present an interesting study on the liquid-liquid phase separation of disease-related alpha-synuclein mutants. The work is quite comprehensive and uses a multi-front approach to analyze the influence of mutations. However, it requires a major revision before it can be considered for publication. I have several suggestions that should be taken into consideration, which would improve the manuscript.
Major points
1. Recent reports have shown that fluorescent labels have a significant effect on the condensation of aSYN. In this manuscript, the authors use YFP, EGFP, mTurquoise, mCherry, mScarlet, mEmerald, Alexa 488. This raises two questions. Why did the authors use such a wide variety of fluorescent labels and did they account for the influence of these protein modifications?
2. Regarding the point about fluorescent labels. The authors should include at least one assay (as supporting information) where they compare the differently labeled aSYN, since the labels may have a significant effect on condensate formation.
3. A30P, A53T mutations have been shown to promote or inhibit aggregation based on the applied experimental conditions. Here, the manuscript states that A53T promotes and A30P reduces condensate formation. It is possible that the effects seen by the authors are condition-dependent and are only observed under their selected conditions. This point should be discussed in more detail.
4. There is a large variety of statistical methods used for each assay, which appears quite strange and makes it difficult to understand the significance in each case. For example, Figure 1 uses “One-Way ANOVA Dunnett’s multiple comparison”, “Two-Way ANOVA Tukey’s multiple comparison, Figure 2 “One-Way ANOVA with Geisser-Greenhouse correction”, Figure 4 “Unpaired two-tailed t-test”. Some figures show error bars as “s.d.”, others as “s.e.m.”. It would be better to standardize the statistical analysis between the different assays.
5. Lines 92 – 93 describe that A53T is highly prone to aggregation, but the condensates did not contain any signs of aggregation during the FRAP assay with hexanediol. What were the time frames for the LLPS and FRAP measurements and did the authors use the exact same incubation times for all the variants? There is a possibility that the variants, which do not form condensates (as described by the authors), simply require a longer incubation period.
6. Protein LLPS typically requires a critical concentration of molecules. Have the authors examined whether the cytosolic concentration of each protein variant is equal? These concentration variations could contribute to the apparent lack of condensates in certain cases.
7. Figure 1 A53T and E83Q in panel D show some signs of increased turbidity at lower PEG and protein concentrations. Were these random anomalies or did these specific conditions result in condensate/aggregate formation?
8. The PEG and protein concentration heat maps can be moved to supporting information, since they do not provide any significant information. It is well established that higher PEG and protein concentrations promote condensation, and the authors used the highest ones for each of their assay.
9. The structure and narrative flow of the manuscript requires some improvements. When reading the text, the sequence of results appeared out of order. For example, Figure 1 shows variants that promote condensation, then Figure 2 displays condensate formation with pS129, followed by Figure 3, which shows variants that reduce condensate formation. Would it not be more logical to describe variants that promote condensation, followed by variants that reduce condensation and then include the additional data with pS129.
Minor points
1. The microscopy images and turbidity heatmaps have large labels (WT, E46K, etc.) within the images. I would suggest placing the labels above the images, to not obscure the shown data.

2. The methods include an Alexa Fluor 594 modification, but there is no such variant in either the main text or supporting information.
3. Figure 1 panel D heatmap with E83Q is missing the X-axis label.
4. Figure 4 panel A labels are not aligned (EGFP, E46K, etc.)
5. Lines 294 – 295 and lines 303 - 304 “n indicates biological repeats”. The figures do not show the n values. Similar problem with other figures, where n values are either not shown or described differently in each case.
6. Figure 1 “Turbidity (O.D. at 350 nm”. Why not simply use O.D. instead of turbidity, since this was the parameter that was measured.
7. Figure 2 panel C does not show p values.
8. Figure 3 and other parts of the text “disease variants reduce condensate formation”. Technically, the variants are less prone towards condensate formation. They themselves do not prevent condensation.
9. Figure 3 panel E Y-axis is too large and can be reduced to 0.0 – 1.0 to better represent the data.
10. Line 539 “CO2” missing a subscript.
11. Line 625 “M-1cm-1” missing superscripts
12. Manufacturer name variation in the method section. For example: “IBIDI”, “ibidi”.

Reviewer #2

(Remarks to the Author)
Chandran et al. present a scientifically rigorous and primarily biochemical investigation of α-synuclein disease-associated variants and their differential capacities to form condensates in vitro—protein-enriched, droplet-like structures with amyloid-like properties that are thought to contribute to pathogenic aggregation. Key findings that advance the field include: (1) the demonstration that the E46K (previously reported) and E83Q (novel) α-synuclein variants enhance condensate formation, whereas A30P, A30G, G51D, and A53E fail to form condensates, and A53T exhibits condensate formation comparable to wild-type; (2) evidence that reduced lipid binding may partially account for the lack of condensate formation observed in the variants that do not undergo this process; and (3) the observation that the presence or absence of condensate formation influences the subcellular localization of α-synuclein in neurons, with variants capable of forming condensates showing enhanced synaptic enrichment.

Overall, this is a well-designed study with conclusions supported by carefully executed and appropriately controlled experiments. The manuscript is clearly written and contributes meaningful mechanistic insight into how disease-linked α-synuclein variants may differ in their biophysical behaviors and potential cellular consequences.

The text would benefit from minor revisions for clarity and consistency:

1) Abstract phrasing: The abstract suggests that both increased and decreased condensate formation contribute to disease, which contrasts with the discussion’s emphasis that these condensates are dynamic and did not form insoluble aggregates under the experimental conditions. Because the manuscript does not directly examine the impact of condensate formation on disease progression, the language in the abstract should be tempered to avoid overstatement.

2) Clarification of experimental context: In the discussion, the statement regarding condensate dynamics and the absence of insoluble aggregates appears based on the HeLa cell model, which was analyzed after only one day of treatment. It would strengthen the accuracy of the interpretation to specify that under the conditions and timeframe of the experiment, insoluble aggregates were not observed.

With these minor revisions, the manuscript will more precisely reflect the scope of the study and its conclusions. Overall, this work provides valuable evidence that deepens our understanding of the molecular mechanisms underlying α-synuclein biology and offers insight into potential cellular processes relevant to Parkinson’s disease pathogenesis.

Version 1:

Reviewer comments:

Reviewer #1

(Remarks to the Author)
The authors have made significant improvements to the manuscript. The current version takes into consideration all the points made during the review and I suggest accepting the manuscript for publication.

Open Access This Peer Review File is licensed under a Creative Commons Attribution 4.0 International License, which permits use, sharing, adaptation, distribution and reproduction in any medium or format, as long as you give appropriate credit to the original author(s) and the source, provide a link to the Creative Commons license, and indicate if changes were made.
In cases where reviewers are anonymous, credit should be given to 'Anonymous Referee' and the source.
The images or other third party material in this Peer Review File are included in the article's Creative Commons license, unless indicated otherwise in a credit line to the material. If material is not included in the article's Creative Commons license and your intended use is not permitted by statutory regulation or exceeds the permitted use, you will need to obtain permission directly from the copyright holder.
To view a copy of this license, visit https://creativecommons.org/licenses/by/4.0/

Point by Point Response to the Reviewers

POINT-by-POINT for Reviewer #1 (Remarks to the Author):

The authors present an interesting study on the liquid-liquid phase separation of disease-related alpha-synuclein mutants. The work is **quite comprehensive and uses a multi-front approach to analyze the influence of mutations.** However, it requires a **major revision** before it can be considered for publication. I have several suggestions that should be taken into consideration, which would improve the manuscript.

Major points

1. Recent reports have shown that **fluorescent labels have a significant effect on the condensation of aSYN.** In this manuscript, the authors use **YFP, EGFP, mTurquoise, mCherry, mScarlet, mEmerald, Alexa 488.** This raises two questions. **Why** did the authors use such a wide variety of fluorescent labels and did they **account for** the influence of these protein modifications?

RESPONSE: For alpha-synuclein (and for other proteins) tagging can have an effect on their protein phase separation and we thank the reviewer for highlighting this point.

All the in vitro work presented in our manuscript has been performed using small molecule labelled aSYN. We used Alexa488 labelled aSYN for imaging experiments, the amount of labelled aSYN was 1% to minimise its influence, having the majority of protein unlabelled. All quantitative turbidity assays were done using 100% un-labelled aSYN. To show that there is no difference when using 1% Alexa488 labelled aSYN, we performed additional experiments which were added as Supplementary Figure S2, comparing 1% Alexa488 labelled aSYN and unlabelled protein in both imaging and turbidity experiments.

All our cell culture experiments used aSYN tagged to YFP or mScarlet to allow live cell imaging. In our HeLa cell work YFP-tagged aSYN was used. All iPSC neuron work used aSYN tagged with mScarlet. A different, red shifted, tag was chosen for the neuronal work, compared to the HeLa cell experiments, to enable studies with additional sensors mostly operating in the green channel. In Supplementary Figure S1, we have added data from additional experiments comparing the effect of YFP and mScarlet tag on condensate formation in HeLa cells. We also performed additional experiments to show that untagged aSYN form condensates in our cell model. For aSYN yeast experiments eGFP was used as a tag as these were established by our collaborator previously.

All other tags mentioned by the reviewer were not used for aSYN but used to label other proteins or markers for co-localization studies. mTurquoise was used to co-label VAMP2. mCherry was used with KDEL and LAMP1 to monitor ER and Lysosome colocalization respectively. mEmerald was used as a tag for synaptophysin to monitor colocalization with aSYN-mScarlet within synapses.

2. Regarding the point about fluorescent labels. The authors should include at least one assay (as supporting information) where they compare the differently labeled aSYN, since the labels may have a significant effect on condensate formation.

RESPONSE: To show that 1% Alexa488 labelled aSYN behaves similar to unlabelled aSYN, we performed additional experiments as requested by the reviewer. These are added as Supplementary Figure S2 and are discussed at the end of paragraph 1 (line 98-111). We performed brightfield imaging experiments for aSYN WT and aSYN E46K, where aSYN either contained 1% Alexa488 labelled aSYN or no labelled protein, using 100% unlabelled aSYN. Further to this we performed additional turbidity measurements, again for aSYN WT and aSYN E46K, showing no difference between 1% labelled aSYN and 100% unlabelled protein.

In Hela cells, we compared the effect of YFP and mScarlet tags and show comparable levels of condensate formation with either tag through live cell imaging. Similar to YFP tagged aSYN E46K, also aSYN E46K-mScarlet shows higher levels of condensate formation compared to WT-mScarlet. Additionally, we also show that untagged WT and E46K undergo VAMP2-induced phase separation. Since unlabelled aSYN cannot be imaged live in cells, cells were fixed and immunostained. This was added as Supplementary Figure S1 and is discussed at the end of paragraph 1 (line 98-111).

3. A30P, A53T mutations have been shown to **promote or inhibit aggregation based on the applied experimental conditions**. Here, the manuscript states that A53T promotes and A30P reduces condensate formation. It is possible that the effects seen by the authors **are condition-dependent and are only observed under their selected conditions**. This point should be discussed in more detail.

RESPONSE: Evaluation of aSYN phase separation in vitro will be dependent on the respective conditions used, i.e. salt concentration, addition of calcium, etc. Our conclusions on phase separation of A53T and lower phase separation of A30P and other variants, have been drawn from experiments performed in cellular systems where conditions resemble a stable environment. Importantly, this was tested using three different model systems. We demonstrate that A30P has no condensation in HeLa cells, no inclusions in yeast, and does not enrich at synapses. For A53T we show that a similar level of phase separation is seen compared to WT, this we observe again in HeLa cells, in yeast, and in iPSC derived neurons where synapse enrichment is seen, but no increase in aSYN synapse size. Aggregation was not directly observed in our model system, which is discussed in more detail in Point 5.

4. There is a **large variety of statistical methods** used for each assay, which appears quite strange and makes it difficult to understand the significance in each case. For example, Figure 1 uses "**One-Way ANOVA Dunnett's multiple comparison", "Two-Way ANOVA Tukey's multiple comparison, Figure 2 "One-Way ANOVA with Geisser-Greenhouse correction**", Figure 4 "**Unpaired two-tailed t-test**". **Some figures show error bars as "s.d.", others as "s.e.m.".** It would be better to standardize the statistical analysis between the different assays.

RESPONSE: This study uses different statistical analyses depending on the respective experimental setup. One-Way ANOVA with Dunnett's multiple comparison was used when one parameter was compared among multiple groups (i.e. different aSYN variants compared to WT). This is the case for experiments showing % of cells forming

condensates or the analysis of synapse enrichment, and has been consistently used throughout, i.e. for Figure 1b, 2b, and 6e-h. A Two-Way ANOVA was used for grouped analysis with 2 variables, i.e. aSYN variant and aSYN concentration as in Fig.1d and Fig.2e. Here a One-Way ANOVA cannot be applied, but a Two-way ANOVA needs to be used. We however, corrected to multiple comparison post-hoc analysis, now also using Dunnett's multiple comparison, comparing each group to WT. An Unpaired two-tailed t-test was used for data, where only two experimental groups are compared. This is the case for Fig.4d, e, i, where conditions with and without lipid are compared, and for Fig. 5c where VAMP2 1-96 and VAMP2 FL are compared. This has been consistently applied throughout the manuscript.

A RM (repeated measure) ANOVA with Geisser-Greenhouse correction has been used for Fig. 3b and 7b for hexanediol experiments, where conditions are compared within the same cell/image before and after treatment. Here the Geisser-Greenhouse correction is a specific recommended correction used for repeated measures. For Fig 3b a Two-Way ANOVA has been performed as multiple groups (WT, E46K, A53T, E83Q) are compared over time. For Fig. 7b a One-Way ANOVA has been performed as only one group (WT) is compared over time.

We carefully checked all statistics and corrected some points. Fig.5c stated One-way ANOVA but is a t-test as only 2 groups are compared. Fig.6g was missing acknowledgment of statistics which have now been added. And Fig.3b needs to state Two-Way RM ANOVA as multiple groups are compared. All figure legends have been checked against the statistical files and have been changed respectively within the manuscript.

Throughout the manuscript we report s.d. for error bars to show the uncorrected error. Only for the FRAP recovery experiments, i.e. Fig 3a in HeLa and 7c/e in iPSC neurons, s.e.m. is shown in order to present curves which can be visually separated.

5. Lines 92 – 93 describe that A53T is highly prone to aggregation, but the condensates did not contain any signs of aggregation **during the FRAP assay with hexanediol**. What were the time frames for the LLPS and FRAP measurements and did the authors use the exact same incubation times for all the variants? There is a possibility that the variants, which do not form condensates (as described by the authors), simply require a longer incubation period.

RESPONSE: The FRAP and hexanediol experiments (Fig. 3a-c) have been performed at the same time point for all variants, at 24 hours. For all variants, including A53T, we have also done FRAP experiments at a longer time point of 48h, however also here no clear drop in FRAP recovery was seen. To clarify this point, we included this information as Supplementary Figure S4, also providing additional analysis of the FRAP recovery. We agree with the reviewer that lower FRAP recovery would be expected, however we thoroughly tested that this is not the case under our experimental conditions.

To ensure that this finding is discussed properly in context, we phrased our discussion more carefully, stating that no aggregation was seen under our specific experimental conditions. Please see lines 252-257.

For the second question, on whether non-condensate variants would form condensates after longer incubation times, we performed additional experiments, evaluating condensate formation for all variants after 48h. For aSYN WT, E46K, A53T and E83Q sustained condensate formation is seen, but no condensate formation is observed for

A30G, G51D, A53E and A30P. This was added as Supplementary Figure S3b and is discussed at the end of paragraph 1 (line 98-111).

6. Protein LLPS typically requires a critical concentration of molecules. Have the authors examined whether the **cytosolic concentration of each protein variant is equal?** These concentration variations could contribute to the apparent **lack of condensates in certain** cases.

RESPONSE: As the reviewer states correctly the phase separation will depend on the respective cytosolic concentration of aSYN. aSYN levels were tested in the iPSC-derived neurons (Supplementary Figure S7), where the respective variants show no significant difference in expression levels. Indeed, the aSYN non-condensate variants show slightly higher levels (G51D, A53E, A30G), speaking against the argument that lower cytosolic levels may account for reduced synapse enrichment. The HeLa cell experiments were performed in transiently transfected cells, therefore cytosolic levels are variable from cell to cell. While the same amount of plasmid DNA was used for each aSYN variant, we now included an additional analysis for aSYN whole cell intensity and cytosolic aSYN intensity. Measurement of aSYN per cell, and measurements of cytosolic levels show that there is no trend towards lower aSYN levels for non-condensate forming variants compared to condensate variants. This was added as Supplementary Figure S3a and is discussed at the end of paragraph 1 (line 98-111).

7. Figure 1 A53T and E83Q in panel D show some signs of **increased turbidity at lower PEG** and protein concentrations. Were these random anomalies or did these specific conditions result in condensate/aggregate formation?

RESPONSE: The indicated increased turbidity measurements are random anomalies. They are seen in the heat map of A53T and E83Q in the 0% PEG conditions where aSYN does not show any condensation. A53T also shows some small increase at a few aSYN concentrations at 5% PEG, however, no increase is seen for 10% PEG, indicating that these again are random anomalies. Furthermore, these variations are mainly contributed from one repeat respectively, further supporting that they are not specific.

8. The **PEG and protein concentration heat maps can be moved to supporting information**, since they do not provide any significant information. It is well established that higher PEG and protein concentrations promote condensation, and the authors used the highest ones for each of their assay.

RESPONSE: We agree that the most prominent changes occur at 15% PEG, however some effect is also seen at 10% PEG at the highest aSYN concentration, i.e. for E46K, which reflects its higher phase separation tendency. To show thorough characterisation and allow evaluation by the reader we still feel that they are relevant to be shown in the main figures. Further to this, the field generally shows full phase diagrams (also including conditions where no condensation is seen, i.e. Ray et al. 2020 Nat Chemistry, Ray et al. 2025 BioRxiv, Röntgen et al. 2025 Sci Adv). We therefore feel that they should be included in the original Figure, however we moved them to represent the last column (Fig. 1e).

9. The **structure and narrative flow of the manuscript requires some improvements**. When reading the text, the sequence of results appeared out of order. For example, Figure 1 shows **variants that promote condensation**, then Figure 2 displays condensate formation **with pS129**, followed by Figure 3, which shows variants that **reduce condensate formation**. Would it not be more logical to **describe variants that promote condensation, followed by variants that reduce condensation and then include the** additional data with pS129.

RESPONSE: We thank the reviewer for raising this point and fully agree that a clear and logical presentation of the results will strengthen the manuscript. In response, we have reorganised both the text and the figures to enhance readability and narrative flow. Figure 1 now presents the condensate-promoting variants, followed by Figure 2, which shows non-condensate variants. Figure 3 consolidates all analyses related to condensate properties, including FRAP and hexanediol experiments, pS129 measurements, which we extended to include staining and quantification for the non-condensate variants, as well as the CD lipid-binding data. This restructuring provides a natural lead-in to Figure 4, in which we examine the relevance of lipid membranes for aSYN phase separation. We hope that these revisions improve the clarity, coherence, and overall readability of the study.

Minor points

1. The microscopy images and turbidity heatmaps have large labels (WT, E46K, etc.) within the images. I would suggest placing the labels above the images, to not obscure the shown data.

We have implemented suggested adjustments and have repositioned labels for the heatmaps and repositioned/changed the size for labels in microscopy images when they obscured data. Thank you for highlighting this issue.

2. The methods include an Alexa Fluor 594 modification, but there is no such variant in either the main text or supporting information.

RESPONSE: The Alexa 594 label relates to secondary antibody staining (Methods lines 693-703). Secondary Alexa594 staining was used for images provided in Figure 3d/g, when pS129 was stained after fixation, and for staining performed on iPSC-derived neurons (Supplementary Figure S6c for tubulin). As these are secondary antibody staining's and not directly attached labels for proteins of interest this is not detailed in the Figures itself.

3. Figure 1 panel D heatmap with E83Q is missing the X-axis label.

RESPONSE: This is correct and has been changed, thanks for highlighting this.

4. Figure 4 panel A labels are not aligned (EGFP, E46K, etc.)

RESPONSE: These have all been aligned again.

5. Lines 294 – 295 and lines 303 - 304 “n indicates biological repeats**”. The figures do not show the n values.** Similar problem with other figures, **where n values are either not shown or described differently in each case**.

RESPONSE: For Figure legend line 294-295, now line 343-346, corresponding to Fig.1b, n relates to the number of biological repeats performed. N is the number of datapoints directly in the graph, i.e. 3 datapoints/biological repeats for all mutants. We changed the colour of the graph to lighter grey, to ensure these are better to see. N is represented the same way throughout, i.e. for Fig.2b, 4d, 4e, 4i and 5c.

For Figure legend line 303-304, now line 350-352, corresponding to Fig.1d, n describes respective independent repeats of turbidity measurements. N is the number of datapoints directly in the graph, i.e. 4 data points/independent repeats for E46K. As these are different kind of experiments n is defined differently.

6. Figure 1 “Turbidity (O.D. at 350 nm”. **Why not simply use O.D. instead of turbidity**, since this was the parameter that was measured.

RESPONSE: This was changed accordingly.

7. Figure 2 panel C does not show p values.

RESPONSE: For Figure 2c, now Figure 3b, showing hexanediol experiments evaluating number of condensates, we now tested significance comparing condensate number before and after hexanediol treatment. Exact p values are detailed in the Figure legend as a different significance value is obtained for each aSYN variant. We also analysed whether there is statistical significance between the variants which is shown in Figure 3c.

8. Figure 3 and other parts of the text “disease variants reduce condensate formation”. Technically, the variants **are less prone towards condensate formation**. They themselves do not prevent condensation.

RESPONSE: We changed the title of Figure 3, now Figure 2, to “aSYN disease variants show decreased condensate formation”, which reflects that they have a lower propensity to phase separate rather than reducing phase separation. We also changed the title of Figure 1 saying “aSYN disease variants show increased condensate formation” rather than “promote condensate formation”.

9. Figure 3 panel E Y-axis is too large and can be reduced to 0.0 – 1.0 to better represent the data.

RESPONSE: In Figure 3e, now Figure 2e, the Y-axis was chosen to be presented with the same scale as in Fig.1d, where variants with higher condensation need to be accompanied. We therefore would like to consider keeping the scale as it currently is, to not overrepresent data of these variants.

10. Line 539 “**CO2” missing a subscript.**

RESPONSE: This was changed accordingly.

11. Line 625 “M-1cm-1” missing superscripts

RESPONSE: This was changed accordingly.

12. Manufacturer name variation in the method section. For example: **“IBIDI”, “ibidi”.**

RESPONSE: This was changed accordingly.

POINT-by-POINT for Reviewer #1 (Remarks to the Author):

Chandran et al. present a scientifically rigorous and primarily biochemical investigation of α-synuclein disease-associated variants and their differential capacities to form condensates in vitro—protein-enriched, droplet-like structures with amyloid-like properties that are thought to contribute to pathogenic aggregation. Key findings that advance the field include: (1) the demonstration that the E46K (previously reported) and **E83Q (novel) α-synuclein variants enhance condensate formatio**n, whereas A30P, A30G, G51D, and A53E **fail to form condensates**, and A53T exhibits condensate formation comparable to wild-type; (2) evidence that **reduced lipid binding may** partially account for the lack of condensate formation observed in the variants that do not undergo this process; and (3) the observation that the presence or absence of condensate formation influences the **subcellular localization of α-synuclein in neurons**, with variants capable of forming condensates showing enhanced synaptic enrichment.

Overall, this is a well-designed study with conclusions supported by carefully executed and appropriately controlled experiments. The manuscript is clearly written and contributes meaningful mechanistic insight into how disease-linked α-synuclein variants may differ in their biophysical behaviors and potential cellular consequences.

The text would benefit from minor revisions for clarity and consistency:

1) Abstract phrasing: The abstract suggests that both increased and decreased condensate formation **contribute to disease**, which contrasts with the discussion's emphasis that these condensates are dynamic and did not form insoluble aggregates under the experimental conditions. Because the manuscript does not directly examine the impact of condensate formation on disease progression, the language in the abstract should be tempered to avoid overstatement.

RESPONSE: The abstract was changed accordingly removing overstatement as suggested now reflecting on the actual work performed. Please see lines 34-36 stating: "Our results emphasize that α-synuclein dysfunction may follow divergent pathways, with both increased and decreased condensate formation."

2) Clarification of experimental context: In the discussion, the statement regarding condensate dynamics and the absence of insoluble aggregates appears based on the HeLa cell model, which was analyzed after only one day of treatment. It would strengthen the accuracy of the interpretation to specify that under the conditions and timeframe of the experiment, insoluble aggregates were not observed.

RESPONSE: To ensure that this finding is discussed properly in context, we phrased our discussion more carefully, stating that no aggregation was seen under our specific experimental conditions. Please see lines 252-257.

With these minor revisions, the manuscript will more precisely reflect the **scope of the study and its conclusions**. Overall, this work provides valuable evidence that deepens our understanding of the molecular mechanisms underlying α-synuclein biology and offers insight into potential cellular processes relevant to Parkinson's disease pathogenesis.
